# Deprescribing of antidepressants: development of indicators of high-risk and overprescribing using the RAND/UCLA Appropriateness Method

**DOI:** 10.1186/s12916-024-03397-w

**Published:** 2024-05-13

**Authors:** Vita Brisnik, Jochen Vukas, Caroline Jung-Sievers, Karoline Lukaschek, G Caleb Alexander, Ulrich Thiem, Petra Thürmann, Cornelius Schüle, Sebastian Fischer, Erika Baum, Michael Drey, Sebastian Harder, Wilhelm Niebling, Ulrike Janka, Olaf Krause, Jochen Gensichen, Tobias Dreischulte, for the POKAL-Group, Peter Falkai, Peter Henningsen, Markus Bühner, Helmut Krcmar, Gabriele Pitschel-Walz, Antonius Schneider, Katharina Biersack, Constantin Brand, Christopher Ebert, Julia Eder, Feyza Gökce, Carolin Haas, Lisa Hattenkofer, Lukas Kaupe, Jonas Raub, Philipp Reindl-Spanner, Hannah Schillok, Petra Schönweger, Clara Teusen, Marie Vogel, Victoria von Schrottenberg, Puya Younesi

**Affiliations:** 1 Institute of General Practice and Family Medicine, LMU University Hospital, LMU Munich, Munich, Germany; 2 Graduate Program “POKAL - Predictors and Outcomes in Primary Care Depression Care”, (DFG - GrK 2621), Munich, Germany; 3 Institute of Medical Data Processing, Biometrics and Epidemiology (IBE), Faculty of Medicine, LMU Munich, Munich, Germany; 4 Pettenkofer School of Public Health, Munich, Germany; 5 Center for Drug Safety and Effectiveness, Johns Hopkins Bloomberg School of Public Health, Baltimore, MD USA; 6 Department of Epidemiology, Johns Hopkins Bloomberg School of Public Health, Baltimore, MD USA; 7 University Medical Center Hamburg-Eppendorf, Hamburg, Germany; 8 Department of Geriatrics, Albertinen-Haus, Hamburg, Germany; 9 Chair of Clinical Pharmacology, Faculty of Health, Department of Medicine, University Witten/Herdecke, Witten, Germany; 10 Philipp Klee-Institute of Clinical Pharmacology, Helios University Hospital Wuppertal, Wuppertal, Germany; 11 Department of Psychiatry and Psychotherapy, LMU University Hospital, LMU Munich, Munich, Germany; 12 Psychiatric Services Lucerne, Lucerne, Switzerland; 13 Institute of General Practice and Family Medicine, Philipps University Marburg, Marburg, Germany; 14 Department of Medicine IV, Geriatrics, LMU University Hospital, LMU Munich, Munich, Germany; 15 Institute for Clinical Pharmacology, University Hospital, Goethe University Frankfurt, Frankfurt, Germany; 16 Department of Medicine, Division of General Practice, Medical Center, University of Freiburg, Freiburg, Germany; 17 Department of Psychiatry and Psychotherapy, Paracelsus Medical University Nuremberg, Nuremberg, Germany; 18 Institute of General Practice and Palliative Medicine, Medical School Hannover, Hannover, Germany

**Keywords:** Antidepressants, Deprescribing, High-risk prescribing, Overprescribing, Adverse drug events

## Abstract

**Background:**

Antidepressants are first-line medications for many psychiatric disorders. However, their widespread long-term use in some indications (e.g., mild depression and insomnia) is concerning. Particularly in older adults with comorbidities and polypharmacy, who are more susceptible to adverse drug reactions, the risks and benefits of treatment should be regularly reviewed. The aim of this consensus process was to identify explicit criteria of potentially inappropriate antidepressant use (indicators) in order to support primary care clinicians in identifying situations, where deprescribing of antidepressants should be considered.

**Methods:**

We used the RAND/UCLA Appropriateness Method to identify the indicators of high-risk and overprescribing of antidepressants. We combined a structured literature review with a 3-round expert panel, with results discussed in moderated meetings in between rounds. Each of the 282 candidate indicators was scored on a 9-point Likert scale representing the necessity of a critical review of antidepressant continuation (1–3 = not necessary; 4–6 = uncertain; 7–9 = clearly necessary). Experts rated the indicators for the necessity of review, since decisions to deprescribe require considerations of patient risk/benefit balance and preferences. Indicators with a median necessity rating of ≥ 7 without disagreement after 3 rating rounds were accepted.

**Results:**

The expert panel comprised 2 general practitioners, 2 clinical pharmacologists, 1 gerontopsychiatrist, 2 psychiatrists, and 3 internists/geriatricians (total *N* = 10). After 3 assessment rounds, there was consensus for 37 indicators of high-risk and 25 indicators of overprescribing, where critical reviews were felt to be necessary. High-risk prescribing indicators included settings posing risks of drug-drug, drug-disease, and drug-age interactions or the occurrence of adverse drug reactions. Indicators with the highest ratings included those suggesting the possibility of cardiovascular risks (QTc prolongation), delirium, gastrointestinal bleeding, and liver injury in specific patient subgroups with additional risk factors. Overprescribing indicators target patients with long treatment durations for depression, anxiety, and insomnia as well as high doses for pain and insomnia.

**Conclusions:**

Explicit indicators of antidepressant high-risk and overprescribing may be used directly by patients and health care providers, and integrated within clinical decision support tools, in order to improve the overall risk/benefit balance of this commonly prescribed class of prescription drugs.

**Supplementary Information:**

The online version contains supplementary material available at 10.1186/s12916-024-03397-w.

## Background

Antidepressants are first-line medications for many psychiatric disorders (including depression, anxiety disorders, and obsessive–compulsive disorder) and have proven to have substantial benefits particularly in patients with moderate to severe symptoms of depression or anxiety disorders [1]. Antidepressants are also some of the most commonly prescribed prescription drugs globally, and their use has increased over time. For example, according to one cross-sectional study in the USA, the proportion of persons aged ≥ 18 years using antidepressants increased by 60% from 6.5 to 10.4% between 1999 and 2010 [2]. More recently, the volume of antidepressant prescribing increased by 97% in England between 2008 and 2018 [3] and by 30% in Germany between 2012 and 2021 [4]. Increased use is desirable if this reflects increased awareness and diagnoses of mental health conditions and reduced stigma associated with affective disorders. However, the increasing use of antidepressants for longer durations than recommended by the guidelines has also been identified as a key driver [5]. General practitioners typically manage maintenance treatment with antidepressants and are therefore often faced with decisions around continuing or deprescribing antidepressants.

While antidepressants play an important role in the pharmacologic management of common and debilitating psychiatric illnesses as well as neuropathic pain and migraine, medication review interventions show they are also used in situations where they may have an unfavorable risk/benefit balance. For example, in one prospective cohort study, antidepressant use could be stopped, reduced, or switched (deprescribed) in almost one-quarter (23.2%) of antidepressant users [6]. Potential indications for stopping antidepressants in primary care include their use in mild forms of depression (where benefits are limited [1, 7, 8]), their long-term use for non-psychiatric illnesses such as primary sleep disorders [9, 10], and excessive treatment durations [5, 11–13]. Newer generation antidepressants (e.g., selective serotonin reuptake inhibitors (SSRIs) and selective serotonin-norepinephrine reuptake inhibitors (SNRIs)) are generally considered safer than traditional ones (e.g., tricyclic antidepressants (TCAs)) [14]. However, even SSRIs and SNRIs are not risk-free, especially among vulnerable older people, where long treatment durations are particularly common [15–17] and where comorbidity and comedication may increase the risk of adverse effects, such as falls and fractures, gastrointestinal bleeding, electrolyte imbalances, and cardiovascular events [18–21]. For example, a recent systematic review shows that antidepressants as a group are associated with a significantly increased risk of falls (odds ratio 1.57 [95% confidence interval (CI) 1.43–1.74]) [20], and in one observational study, the 1-year numbers needed to harm for fractures were 247 (for SSRIs) and 308 (for TCAs) among 65 to 74-year-olds, and 53 and 81 for people 75 years or older, respectively, while mirtazapine only significantly increased fracture risk among the older age group [22].

Despite the opportunities to improve the overall risk/benefit balance of antidepressant use in clinical practice, such opportunities may easily be overlooked by primary care clinicians due to competing priorities. The explicit criteria could help alert prescribers to *consider* deprescribing where indicated, even when *decisions* to deprescribe require considerations of patient-specific balance of benefits and risks as well as patient preferences. In addition to discontinuing antidepressants, deprescribing may also encompass dose reduction or switching to a safer agent, which may be the preferred option if antidepressant therapy continues to be necessary to control symptoms. Although existing generic lists of potentially inappropriate medication (PIM) generally advise caution in the use of antidepressants in the elderly [23], more specific advice as to when deprescribing of antidepressants should be considered is desirable to guide the identification of deprescribing opportunities. As an aid to encourage antidepressant deprescribing where indicated, the aim of this study was to establish evidence-based expert consensus on situations, where a critical review of antidepressant continuation would be warranted in primary care. We envisioned that by prompting earlier and proactive reviews of antidepressant use, the resulting set of explicit criteria could help prevent antidepressant-related harm, especially in vulnerable older people.

## Methods

### Study design

We used a consensus process based on the RAND/UCLA (University of California) Appropriateness Method (RAM) [24] to develop our indicators. First, we assembled a list of candidate indicators based on a structured literature review including primary and secondary English and German literature sources. The candidate indicators were subjected to a three-round expert consensus process, with feedback and synchronous discussion of first and second round ratings before second and third round ratings were placed, respectively.

### Selection of the expert panel

We recruited a diverse set of experts with clinical or scientific experience in the use of antidepressants from different fields of professional practice in order to achieve a broad range of perspectives and expertise. We therefore recruited general practitioners, psychiatrists, geriatricians, a gerontopsychiatrist, and clinical pharmacologists from Germany. We identified an initial set of 20 potential experts using our professional networks, planning for the ultimate inclusion of approximately 12 participants. Experts participating in the consensus process did not receive any compensation for their participation.

### Identification of candidate indicators

#### Definitions

For the purposes of this study, we distinguished between two types of settings, where antidepressant deprescribing should be considered. We defined high-risk prescribing as the use of antidepressants in the presence of risk factors increasing the likelihood of an adverse drug reaction (ADR), whether comedication (drug-drug interactions), comorbidities (drug-disease interactions), or advanced age (drug-age interactions). We defined overprescribing as the use of antidepressants for longer periods than indicated or for indications without evidence of relevant benefit or at higher doses than indicated. We included SSRIs, SNRIs, TCAs, monoamine oxidase inhibitors (MAOIs), and atypical antidepressants such as mirtazapine, trazodone, bupropion, agomelatine, and opipramol in this study. Structurally, opipramol belongs to the class of TCAs and is widely prescribed in Germany for insomnia.

#### High-risk prescribing

In order to identify candidate indicators of high-risk prescribing of antidepressants, we initially searched for previously developed indicators targeting potentially inappropriate antidepressant prescribing [25–29]. We also considered systematic and clinical reviews of adverse antidepressant effects as well as clinical practice guidelines in English and German language. Based on consensus among a subset of co-authors (T.D. and V.B.), we prioritized ADRs for which a continuation of antidepressant use could either lead to serious harm, such as hospital admission, or severely affect patients’ quality of life. We conducted further searches in PubMed/MEDLINE and EMBASE to identify candidate indicators linked to each ADR of interest. To this end, we conducted searches including carefully selected (MeSH and non-Mesh) terms for each specific adverse drug reaction of interest and combined these with terms for each group of antidepressants (e.g., SSRIs). We initially searched for recent systematic reviews and meta-analyses but also considered primary literature where reviews were not available or required updating. If applicable, we also examined the reference lists of important reviews for additional studies. We provide more details of the literature search and the search terms used in Additional file 1.

#### Overprescribing

In order to identify candidate indicators of overprescribing of antidepressants, we considered clinical practice guidelines in English and German languages for depression, anxiety and panic disorders, insomnia, and pain [30–33]. We searched for recommendations concerning treatment duration and the recommended doses when prescribed for insomnia and pain. In addition, we also searched for clinical guideline recommendations (e.g., for dementia) specifically not recommending antidepressants for a first depressive episode.

### Design of the rating form and supporting materials

Members of the expert panel were sent the following materials: the rating form, a summary of clinical evidence summary, and rating instructions. The rating form included the candidate indicators, which were organized into 2 sections (high-risk and overprescribing), and each section was divided into chapters. In the high-risk prescribing section, there were 23 chapters for candidate indicators relating to each ADR (e.g., fall, GI bleeding), while in the overprescribing section, there was 1 chapter for candidate indicators relating to each indication (depression, anxiety, insomnia, pain). The indicators followed a standardized format and were designed as variations around the same topic in order to determine thresholds beyond which a critical review would be considered necessary (1 example is provided in Table 1). For each chapter, we developed a summary of clinical evidence supporting the candidate indicators to be considered by the expert panel as part of the rating process. The rating instructions defined rating constructs and assumptions and provided guidance on how the rating form was to be completed and returned.

**Table 1:** Examples of candidate indicators^a^ linked to falls/fall injuries

Candidate indicators	Median “necessity” rating after rating round 2	Accepted for the 3rd round
**ADR: falls and fall-related injuries**		
A. History of fall and prescribed one single antidepressant with sedating, anticholinergic, or orthostatic properties (TCA, mirtazapine, or trazodone)	7	Accepted
B. History of fall and prescribed one single antidepressant with sedating, anticholinergic, or orthostatic properties (TCA, mirtazapine, or trazodone) with one further fall risk-increasing drug	8	Redundant
C. History of fall and prescribed one single antidepressant with sedating, anticholinergic, or orthostatic properties (TCA, mirtazapine, or trazodone) with two or more further fall risk-increasing drugs	9	Redundant

^a^Multiple variations of candidate indicators were rated in order to identify thresholds beyond which a critical review of antidepressant use was considered necessary. Candidate indicators B and C were found to be redundant after candidate indicator A was accepted (necessity rating of ≥ 7)

We piloted the rating form, the summary of clinical evidence, and the supporting instructional materials with one psychiatrist, one clinical pharmacologist, and one general practitioner, using their feedback to optimize the final version of the first round survey. All materials are available from the authors upon request.

### Rating constructs and scales

Each expert rated each candidate indicator based on a 9-point Likert scale representing the necessity of a critical review of that particular clinical instance (1 to 3 = not necessary; 4 to 6 = might be necessary; 7 to 9 = clearly necessary). We also asked experts to rate the subset of indicators reflecting high-risk prescribing for “likelihood of harm,” and each linked ADR was additionally rated for “severity of harm.” For all candidate indicators, the necessity to review was the decisive criterion for the acceptance of indicators, and we used these latter ratings to inform discussion in case of disagreements.

#### Necessity of review

We asked for the necessity of review rather than the necessity of deprescribing since deprescribing decisions may depend on a patient-specific balance of benefits and risks as well as patient preferences, which are unfeasible to pre-specify. We defined “critical review” as a critical assessment of the balance of benefits and risks of antidepressant use to be conducted within 3 months, which would involve patient empowerment and shared decision-making and take at least 30 min to conduct. A critical review may result in dose reduction, switching, or discontinuation of an antidepressant (deprescribing). Consistent with RAM, we defined “necessary” to mean that omitting the review would be considered improper care, that conducting the review would have a reasonable chance of benefitting the patient and that the benefit is not small (Table 2).

**Table 2:** Rating constructs, definitions, and rating scales used in all three rounds of expert panel ratings

Rating construct	Definition and rating scales
Necessity of review	For an average patient treated with antidepressants in primary care: Assuming no overprescribing/high-risk prescribing, how necessary^a^ is it to conduct a critical review* of antidepressant use within the next 3 months in order to prevent adverse effects/reduce medication burden? 1–3 = not necessary; 4–6 = might be necessary; 7–9 = clearly necessary
Likelihood of harm	How likely is it that the patient will experience an adverse drug reaction if the clinical situation was to be continued for another year? 1–3 = unlikely; 4–6 = possible; 7–9 = probable
Severity of harm	If the patient experienced an adverse drug event as a result of antidepressant use, how severe would it be? 1–3 = minor; 4–6 = moderate; 7–9 = major

^a^See the “Methods” section for further detail regarding the definitions of a critical review and the rating construct of necessary

#### Likelihood and severity of harm

We defined *likelihood of harm* as the likelihood of the adverse drug reaction happening if the clinical situation was to be continued for another year and *severity of harm* as the severity of the harm if the adverse drug events happened as a result of antidepressant use.

#### Rating scales

We used ordinal scales of 1 to 9 for all ratings. We pre-specified that an indicator would be accepted as *necessary*, when the median across all expert assessments was ≥ 7, and there was no disagreement. Disagreement was pre-specified to mean that at least 30% of the experts rated items 1–3, and at least 30% rated items 7–9. Candidate indicators with a median of < 7 or disagreement were rejected.

### RAM process

The RAM process comprised two virtual discussions and three rating rounds. All expert panel members were sent the first RAM survey by e-mail (on 01/08/2022), together with a one-page overview of the project, rating instructions, and the summarized clinical evidence for each overarching topic. Experts were instructed to place their ratings based on both the evidence report and clinical judgment. The experts were instructed to place their ratings in relation to an average patient on antidepressants treated in primary care. The panel members were given 4 weeks to complete the first round of the RAM survey.

The experts met in a moderated videoconference (moderated by TD) on 01/09/2022. The first round assessments were summarized and presented to the experts, highlighting the median and distribution of ratings as well as the presence of disagreement. The focus of the videoconference was the discussion of indicators with disagreement for the necessity ratings after the first round assessment. After discussing the candidate indicators relating to each ADR (in case of high-risk prescribing indicators) or each indication (in case of overprescribing indicators), the panel members had time to complete the second round assessment.

Indicators reaching a median of ≥ 7 after the second round of assessment were summarized, and the redundant indicators were removed (see Table 1 for an example). The pre-final list of indicators was sent to expert panel members on 24/02/2023. The experts met on 16/03/2023 for a second virtual discussion. The summarized list of indicators allowed the experts to discuss the remaining indicators in more detail and if necessary optimize them for implementation in primary care. Requests for changes in the indicators were implemented and put to a final vote in a third rating round using the same rating constructs and scales as before.

## Results

### Expert panel composition

The first round RAM survey was sent to 11 expert panel members. All 11 experts participated in the moderated videoconference, and 10 (90.9%) members successfully completed the second and third round survey (general practitioners (*n* = 2), clinical pharmacologists (*n* = 2), psychiatrists (*n* = 2), geriatricians (*n* = 3), and a gerontopsychiatrist (*n* = 1)). All 10 experts were clinically trained physicians (with an average [range] of 30 [13 to 46] years since training) with regular patient care experience, and 9 (90.0%) also had current research experience.

### Candidate indicators

#### High-risk prescribing

The literature search identifying potential candidate indicators yielded a recent systematic review that contained an extensive list of potential prescribing safety indicators related to mental health [34]. Antidepressant-associated indicators from this review were combined with those included in commonly used PIM lists [25–29]. Further high-risk prescribing candidate indicators were identified from clinical practice guidelines, such as those for depression or chronic heart failure [30, 35], literature reviews of adverse events associated with antidepressant drugs [14, 36–38], and further reviews from searches for selected ADRs (detailed in Additional file 1). The first round of the survey included 212 variations of potential candidate indicators for high-risk prescribing. It should be noted that many indicators were highly dose-specific, e.g., experts were asked to differentiate between the risk of different dose levels of TCAs per day and also between the risk of synergistic pharmacological effects combining 2 or more drugs (e.g., with anticholinergic properties). This allowed for a very fine differentiation between potentially high-risk constellations.

#### Overprescribing

For depression and anxiety, the indicators of overprescribing focused mainly on the duration of treatment without symptom improvement or on the total duration of treatment. With the exception of doxepin, antidepressants are not officially approved for insomnia, and guidelines are not clear on dose recommendations or duration of treatment for antidepressants as a sedative [32]. Dose recommendations were also considered for pain [33]. The first round of the survey included 70 variations of potential candidate indicators for overprescribing.

### RAM process

#### High-risk prescribing

**Fig. 1:**
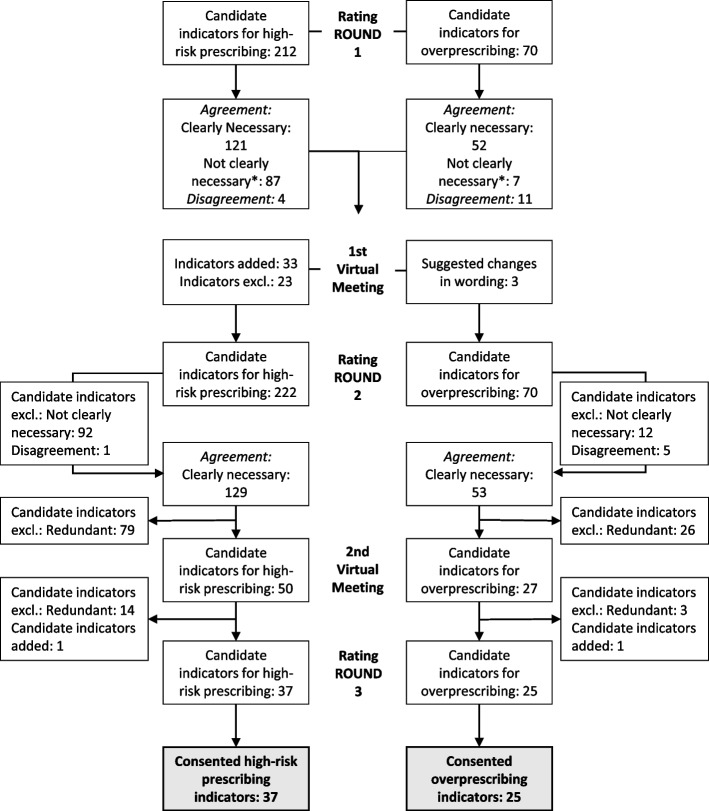
Flow chart showing the RAM process. *Not clearly necessary: might be necessary 4 to 6 or not necessary 1 to 3

Figure 1 shows that after round 1, 121 (57.1%) of 212 candidate indicators were accepted as “clearly necessary to review.” Six indicators (2.8%) were consented as “not necessary” and 81 indicators (38.2%) as “might be necessary to review.” There was disagreement for 4 indicators (1.9%). Changes after the first round assessment and during the moderated videoconference resulted in 222 potential high-risk prescribing indicators being rated in the second round, of which 129 candidate indicators (58.1%) were accepted as “clearly necessary,” 6 indicators (2.7%) as “not necessary,” and 86 indicators (38.7%) as “might be necessary to review.” There was disagreement for 1 indicator (0.5%). We provide the expert ratings of round 2 in Additional file 2. Removing redundant candidate criteria yielded 50 indicators for high-risk prescribing. After the second moderated videoconference, 37 remaining indicators were validated in the third round of assessment, and all were agreed to be “clearly necessary to review.” Changes to the indicators after the second round of assessment and the rationale for the changes are detailed in Additional file 3. Table 3 reports the consented indicators after the third round of assessment. Prioritized indicators target patients who are particularly vulnerable to (risk factors: drug-drug, drug-disease, or drug-age interactions) or who have developed adverse drug reactions. High-risk prescribing indicators included constellations of known anticholinergic (e.g., cognitive decline, delirium, constipation, voiding disorders, and glaucoma) and cardiovascular (e.g., QTc prolongation) risks but also falls, orthostatic hypotension/dizziness, bleeding, serotonin syndrome, hyponatremia, hepatic injury, sleep disturbances, and sexual dysfunction. Some of these constellations could lead to serious harm, if antidepressants are continued, particularly in older adults with comedication and comorbidities (e.g., cardiovascular adverse effects, fall-related injuries, delirium, gastrointestinal and intracranial bleeding, hyponatremia). The remaining constellations with the corresponding adverse drug reactions can severely affect patients’ quality of life (constipation, sleep disturbances, and sexual dysfunction). Indicators with the highest ratings (median = 9) included those suggesting the possibility of cardiovascular risks such as QTc prolongation associated with citalopram and escitalopram, delirium associated with anticholinergic antidepressants, gastrointestinal bleeding associated with SSRIs and SNRIs, and liver injury associated with agomelatine.

**Table 3:** Summary of the final indicators of high-risk prescribing with median ratings of 7 to 9 on the necessity to review without disagreement

High-risk prescribing indicators	Median	Agreement	Range
**A. Cardiovascular adverse effects**			
1. Prescribed SNRI, TCA (in doses ≥ 50 mg/day)^A^, or tranylcypromine^B ^- and the patient has a history of chronic heart failure	8	90%	6–9
2. Prescribed TCA (in doses ≥ 50 mg/day) - and the patient has a history of ischemic heart disease	8	100%	7–9
3. Prescribed > 20 mg citalopram or > 10 mg escitalopram daily - and the patient is aged ≥ 65 years (risk of QTc prolongation)	7	70%	2–9
4. Prescribed citalopram and escitalopram - and the patient has long QT syndrome or is at risk of long QT syndrome (e.g., (advanced) chronic heart failure, ischemic heart disease, myocardial hypertrophy, bradyarrhythmias, or an ongoing risk of hypokalaemia^c^)	9	100%	7–9
5. Prescribed citalopram, escitalopram, or TCA (in doses ≥ 50 mg/day) - and the patient is co-prescribed ≥ 1 further drug with any risk of TdP^c^	7	78%	6–9
6. Prescribed TCA (in doses ≥ 50 mg/day), SNRI, bupropion, or tranylcypromine^B ^- and the patient has developed tachycardia	8	90%	6–9
7. Prescribed fluoxetine, paroxetine, or bupropion - and the patient is co-prescribed metoprolol or propranolol (risk of bradycardia)	7	60%	2–9
8. Prescribed SNRI, TCA (in doses ≥ 50 mg/day), bupropion, or tranylcypromine - and the patient has uncontrolled hypertension^c^	8	100%	7–9
9. Prescribed SNRI, TCA (in doses ≥ 50 mg/day), bupropion, or tranylcypromine - and achieving hypertension control requires ≥ 3 antihypertensive drugs	8	80%	4–9
**B. Orthostatic hypotension (OH)/dizziness**			
1. Prescribed TCA (in doses ≥ 50 mg/day), trazodone, or tranylcypromine - and the patient has developed persistent OH/dizziness under treatment	8	100%	7–9
2. Prescribed SSRI, SNRI, or mirtazapine - and the patient is aged ≥ 65 years and has developed persistent OH/dizziness under treatment	8	100%	7–9
3. Prescribed TCA (in doses ≥ 50 mg/day), trazodone, or tranylcypromine - and the patient is aged ≥ 65 years and co-prescribed ≥ 1 further drug with known blood pressure lowering effect (e.g., α-blockers, β-blockers, nitrates, SGLT inhibitors, levodopa, antipsychotics)^c^	7	60%	5–9
4. Prescribed SSRI, SNRI, or mirtazapine - and the patient is aged ≥ 65 years and co-prescribed ≥ 2 further drugs with blood pressure lowering effect (e.g., α-blockers, β-blockers, nitrates, SGLT inhibitors, levodopa, antipsychotics)	7	67%	5–9
**C. Falls and fall-related injuries**			
1. Prescribed any antidepressant - and the patient is aged ≥ 65 years and co-prescribed ≥ 1 further fall risk-increasing drug^c^	7	60%	2–9
2. Prescribed any antidepressant - and the patient has a history of falls	8	60%	2–9
3. Prescribed any antidepressant - and the patient has cognitive impairment or dementia	7	60%	2–9
4. Prescribed any antidepressant - and the patient has a history of stroke and co-prescribed ≥ 1 further fall-risk-increasing drug	8	70%	2–9
**D. Cognitive decline and delirium**			
1. Prescribed anticholinergic antidepressant opipramol, other TCAs (in doses ≥ 50 mg/day), or paroxetine - and the patient has cognitive impairment or dementia	8	90%	6–9
2. Prescribed anticholinergic antidepressant opipramol, other TCAs (in doses ≥ 50 mg/day), or paroxetine - and the patient has a history of delirium and co-prescribed ≥ 1 further drug known to induce delirium (e.g., benzodiazepines, opioids, antihistamines, diuretics)^c^	8	100%	7–9
3. Prescribed anticholinergic antidepressant opipramol, other TCAs (in doses ≥ 50 mg/day), or paroxetine - and the patient is aged ≥ 65 years and co-prescribed ≥ 2 further drugs known to induce delirium (e.g., benzodiazepines, opioids, antihistamines, diuretics)	9	100%	7–9
**E. Serotonin syndrome**			
1. Prescribed tranylcypromine - and the patient is co-prescribed ≥ 1 further serotonergic drug (e.g., tramadol, fentanyl, triptans, metoclopramide, SSRI, SNRI, TCA)^c^	7	70%	5–9
2. Prescribed SSRI, SNRI, or TCA (in doses ≥ 50 mg/day) - and the patient is co-prescribed ≥ 2 further serotonergic drugs other than tranylcypromine (e.g., tramadol, fentanyl, triptans, metoclopramide, another serotonergic antidepressant)	8	80%	5–9
**F. Gastrointestinal bleeding**			
1. Prescribed SSRI or SNRI - and the patient is aged ≥ 65 years and co-prescribed a single of the following without GI protection: antiplatelet, anticoagulant, and NSAID	7	60%	6–9
2. Prescribed SSRI or SNRI - and the patient is aged ≥ 65 years and co-prescribed ≥ 2 of the following: antiplatelet, anticoagulant, and NSAID (regardless of GI protection)	8	100%	7–9
3. Prescribed SSRI or SNRI - and the patient has at least one risk factor for GI bleeding (history of peptic ulcer disease, GI bleeding, or hemophilia) and co-prescribed ≥ 1 of the following: antiplatelet, anticoagulant, and NSAID (regardless of GI protection)	9	70%	2–9
**G. Bleeding**			
1. Prescribed SSRI - and the patient has a history of a bleeding event and co-prescribed ≥ 1 of the following: anticoagulant or antiplatelet	8	70%	6–9
2. Prescribed SSRI - and the patient has at least one risk factor for intracranial bleeding (aged ≥ 65 years, history of stroke, history of dementia) and co-prescribed ≥ 1 of the following: anticoagulant or antiplatelet	7	90%	5–9
**H. Constipation**			
1. Prescribed anticholinergic antidepressant opipramol, other TCAs (in doses ≥ 50 mg/day), or paroxetine - and the patient has persistent constipation	7	70%	5–9
2. Prescribed anticholinergic antidepressant opipramol, other TCAs (in doses ≥ 50 mg/day), or paroxetine - and the patient is aged ≥ 65 years and co-prescribed ≥ 2 further drugs known to have constipating effects (e.g., calcium antagonists, opioid, antihistamines, antipsychotics)	8	90%	5–9
**I. Hyponatremia**			
1. Prescribed any antidepressant - and the patient has developed hyponatremia (< 130 mmol/l) under treatment without being treated with a diuretic	7	90%	6–9
2. Prescribed SSRI or SNRI - and the patient is aged ≥ 65 years and co-prescribed ≥ 2 further drugs known to cause hyponatremia (e.g., (thiazide) diuretics, antipsychotics, anticonvulsants, proton pump inhibitors)^c^	8	80%	2–9
**J. Hepatic injury**			
1. Prescribed agomelatine - and the patient has developed elevated serum transaminase levels (> 3 times the upper normal range) under treatment	9	90%	6–9
2. Prescribed agomelatine - and the patient has a hepatic impairment (i.e., cirrhosis or active liver disease)	8	80%	
**K. Voiding disorders**			
1. Prescribed anticholinergic antidepressant opipramol, other TCAs (in doses ≥ 50 mg/day), or paroxetine - and the patient has a history of voiding disorders (e.g., urinary retention or benign prostatic hyperplasia) or has developed urinary retention under treatment	7	60%	3–9
**L. Glaucoma**			
1. Prescribed anticholinergic antidepressant opipramol, other TCAs (in doses ≥ 50 mg/day), or paroxetine - and the patient has a history of angle closure glaucoma or has developed angle closure glaucoma under treatment	8	60%	6–9
**M. Sleep disturbances/agitation**			
1. Prescribed SSRI, SNRI, MAOI, or bupropion - and the patient has persistent sleeping disturbances (e.g., insomnia, restless leg syndrome) or is experiencing agitation	7	90%	6–9
**N. Sexual dysfunction**			
1. Prescribed SSRI or SNRI - and the patient has developed sexual dysfunction	8	90%	6–9

*SSRI* selective serotonin reuptake inhibitors, *SNRI* selective serotonin-norepinephrine reuptake inhibitors, *TCA* tricyclic antidepressant, *TdP* torsades de pointes, *NSAID* nonsteroidal anti-inflammatory drugs, *GI* gastrointestinal, *MAOI* monoamine oxidase inhibitors

^a^It cannot be excluded that low-dose TCAs also have significant adverse effects, as evidence of the safety of low-dose TCAs is sparse

^b^Especially when co-administered with tyramine-containing food

^c^See Additional file 3 for further details regarding the definitions and further examples of comedication

#### Overprescribing

Fig. 1 shows that after round 1, 52 (74.3%) of 70 candidate indicators were accepted as “clearly necessary to review.” One indicator (1.4%) was consented as “not necessary” and 6 indicators (8.6%) as “might be necessary to review.” There was disagreement for eleven indicators (15.7%). A total of 53 candidate indicators (75.7%) were accepted as “clearly necessary,” 0 indicators (0%) as “not necessary,” and 12 indicators (17.1%) as “might be necessary to review” in the second round of assessment. There was disagreement for 5 indicators (7.1%). We provide the expert ratings of round 2 in Additional file 4. Removing redundant candidate criteria yielded 27 indicators for overprescribing. After the second moderated videoconference, 25 remaining indicators were validated in the third round of assessment, and all were agreed to be “clearly necessary to review.” Table 4 reports the consented indicators after the third round of assessment. Prioritized indicators target patients who have a high medication burden potentially associated with antidepressants due to long treatment durations, inappropriate indications, or high doses.

**Table 4:** Summary of final indicators of overprescribing with median ratings of 7 to 9 on the necessity to review without disagreement

Overprescribing indicators	Median	Agreement	Range
**Depression**			
1. Prescribed an antidepressant - and the patient has a first episode of mild depression	8	70%	3–9
2. Co-prescribed two antidepressants - and the patient has a first episode of moderate depression	8	67%	3–9
3. Prescribed an antidepressant in monotherapy for ≥ 4 weeks - and the patient is aged < 65 years with no signs of clinically relevant symptom improvement^1^	7	80%	4–9
4. Prescribed an antidepressant in monotherapy for ≥ 6 weeks - and the patient is aged ≥ 65 years with no signs of clinically relevant symptom improvement^1^	9	90%	6–9
5. Prescribed an antidepressant in monotherapy - and the patient has previously used two or more different antidepressants (inadequate response)	7	70%	3–9
6. Prescribed an antidepressant in monotherapy, combination, or augmentation > 12 months for a first episode of moderate or severe depression - and the patient has achieved full remission	7	80%	3–9
7. Prescribed an antidepressant in monotherapy, combination, or augmentation > 2 years with a history of 2 or more depressive episodes with functional impairment in the last 5 years - and the patient has achieved full remission	7	70%	4–9
8. Prescribed SSRI at a dose of > 1 DDD - and the patient has no clinically relevant symptom improvement under an SSRI dose ≤ 1 DDD (no further dose increase if symptoms remain/worsen)	8	70%	3–9
9. Prescribed two antidepressants **- **and none of those is mirtazapine, mianserin, or trazodone	8	90%	6–9
**Anxiety**			
1. Prescribed an antidepressant for ≥ 8 weeks - and the patient is aged < 65 years with no signs of clinically relevant symptom improvement^1^	8	90%	6–9
2. Prescribed an antidepressant for ≥ 12 weeks - and the patient is aged ≥ 65 years with no signs of clinically relevant symptom improvement	8	100%	7–9
3. Prescribed an antidepressant > 12 months for anxiety - and the patient has achieved full remission	7	70%	2–9
4. Prescribed an antidepressant for anxiety - and the patient is co-prescribed benzodiazepine > 4 weeks	9	100%	7–9
**Insomnia**			
1. Prescribed TCA ≥ 50 mg/day for insomnia^2 ^- and the patient has no other indications for an antidepressant	7	70%	5–9
2. Prescribed trazodone ≥ 50 mg/day for insomnia - and the patient has no other indications for an antidepressant	8	80%	5–9
3. Prescribed mirtazapine ≥ 30 mg/day for insomnia - and the patient has no other indications for an antidepressant	7	80%	3–9
4. Prescribed a sedating antidepressant > 8 weeks for insomnia - and the patient has no other indications for an antidepressant	8	80%	5–9
**Pain**			
1. Prescribed a TCA ≥ 75 mg/day for neuropathic pain - and the patient has no other indications for an antidepressant	7	60%	3–9
2. Prescribed venlafaxine ≥ 150 mg/day for neuropathic pain - and the patient has no other indications for an antidepressant	8	80%	6–9
3. Prescribed SSRI or mirtazapine for neuropathic pain - and the patient has no other indications for an antidepressant	8	90%	6–9
4. Prescribed any antidepressant for non-specific low back pain - and the patient has no other indications for an antidepressant	8	90%	6–9
5. Prescribed TCA or SNRI as analgesic for pain (e.g., pain other than neuropathic pain, tension headache, migraine, or fibromyalgia syndrome) - and the patient has no other indications for an antidepressant	8	70%	5–9
**Miscellaneous**			
1. Prescribed any antidepressant - and the patient has chronic heart failure and a first episode of mild or moderate depression	7	70%	2–9
2. Prescribed any antidepressant - and the patient has dementia and a first episode of mild or moderate depression	7	70%	2–9
3. Prescribed agomelatine - and the patient is aged ≥ 75 years	7	70%	5–9

*DDD* defined daily dose, *SSRI* selective serotonin reuptake inhibitors, *SNRI* selective serotonin-norepinephrine reuptake inhibitors, *TCA* tricyclic antidepressant

^1^At the maximum tolerated or recommended dose

^2^Irrespective of the length of the treatment

## Discussion

### Summary of findings

Antidepressants are some of the most commonly prescribed drugs in the world. Despite their value, there are instances where they may have an unfavorable risk/benefit balance. We performed a structured literature review and expert consensus process (RAM) in order to synthesize and reach consensus on a set of 62 explicit indicators (37 indicators of high-risk prescribing and 25 indicators of overprescribing of antidepressants) that should prompt a critical review of antidepressant continuation. Indicators with the highest ratings included those suggesting the possibility of cardiovascular risks such as QTc prolongation, delirium, gastrointestinal bleeding, and liver injury associated with certain antidepressants in specific patient subgroups with additional risk factors.

### Comparison to literature

To the best of our knowledge, this is the first consensus study focused on identifying indicators for high-risk and overprescribing of antidepressants. Compared to more generic lists of potentially inappropriate medications [23, 39], our focus on a specific class of drugs allowed for the development of a comprehensive set of indicators specifically related to antidepressants. For example, STOPP (Screening Tool of Older Person’s Prescriptions/START (Screening Tool to Alert doctors to Right Treatment) version 3 includes 10 indicators related to antidepressants (7.5%) [23], while FORTA (Fit fOR The Aged) identifies individual antidepressants for 6 indications [39]. In comparison, this study identified 37 high-risk prescribing indicators related to a broad spectrum of adverse outcomes. Our findings also include certain risks that are inconsistently listed in clinical guidelines, such as bleeding and fall risks associated with SSRIs, despite systematic reviews supporting these risks [18, 20].

Although broadly consistent with previously published tools for identifying PIMs [23], some differences are worth highlighting. First, the indicator set developed here is likely to identify more patients at risk of bleeding. For example, in contrast to the STOPP criteria, our set also considers the bleeding risk associated with SNRIs [40, 41] as well as co-prescription with nonsteroidal anti-inflammatory drugs (NSAIDs) and/or antiplatelets [40, 42]. Second, in contrast to STOPP Fall, our expert panel did not confirm a higher fall risk for tricyclic antidepressants than other antidepressants [43], and our set identifies additional patients at risk for falls, such as those with cognitive impairment or dementia. Third, our set identifies a particular need to review antidepressants in patients with hyponatremia who are not co-prescribed diuretics (which would then primarily require review) and also accounts for the co-prescription of antidepressants with other hyponatremia-inducing drugs. Fourth, unlike previously published lists [23], our indicator set considers the risk of insomnia with activating antidepressants (such as SSRIs, SNRIs, MAOIs, or bupropion). Fifth, our indicators also identify antidepressant risks related to serotonin syndrome, hepatic injury, and sexual dysfunction, which are usually not included in PIM lists as they are not unique to older adults. Several factors may contribute to these differences, including our focus on identifying patients in need of a review specifically targeting antidepressants, the composition of our expert panel, and the evolution of clinical evidence.

### Strengths and limitations

Our study has several strengths. First, an important advantage of the RAM compared to the commonly used Delphi process is that panelists have the opportunity to exchange perspectives in between rounds and for moderators to ensure that rating constructs are understood correctly and applied consistently. Second, our expert panel included generalists and specialists that promoted informed discussions regarding how to optimally balance comprehensiveness, relevance, and feasibility of implementation in primary care. Third, our indicators present a more holistic view of the patient and his or her individual situation combining patient-specific risk factors (e.g., certain comorbidities, co-prescribed medications). Moreover, pharmacological features such as dose-related and synergistic effects were taken into account. While the experts practiced in Germany, our literature review and supporting evidence base were comprehensive and international in scope. Although we cannot exclude that the selection and wording of candidate indicators may have influenced our findings, all experts were given an opportunity to suggest additional indicators and clarify ambiguous wording during panel meetings. Our indicator set focuses on a broad set of adverse effects and common indications for antidepressant use, but it is important to note that it cannot cover all instances of overprescribing or sources of antidepressant-related adverse events.

### Implications for clinical practice and research

The indicators consented in this study may be used to inform clinical practice as well as clinical surveillance and research. Clinical practice guidelines typically focus on the appropriate use of antidepressants but do not explicitly state when their use may require caution or review with a view to deprescribing. This set of indicators may therefore complement such guidelines and could be used in conjunction with other established PIM lists [23, 39, 44]. Decision aids, ideally implemented in practice management systems, can trigger a process of shared decision-making, thereby strengthening the physician–patient interaction, ensuring desired effects, and preventing adverse effects of antidepressants before they occur. Indicators could also be used as a decision aid prior to starting antidepressants, but this may not be sufficient given that patients’ clinical circumstances may change during treatment. The indicators could also be used to monitor antidepressant use at the population level and as endpoints to evaluate the impact of interventions to enhance the appropriate use of antidepressants in primary care. The indicators may also be useful in informing and empowering patients, which may be particularly relevant in disjointed health care systems, where changes in comorbidity and comedication that could unfavorably affect the benefit/risk ratio of antidepressant use may remain unnoticed by the antidepressant prescriber. However, providing detailed information about potential risks must be balanced against the risk of adversely affecting patient adherence.

In addition, it is important to note that despite its potential benefits, deprescribing antidepressants implies a risk of disease recurrence and withdrawal symptoms. The risk of the latter can be reduced by close monitoring and timely adaptation of tapering schemes, but their implementation may be time-consuming to clinicians and patients alike. The indicators developed here may therefore only serve as a prompt to consider deprescribing, but whether deprescribing should be attempted (or whether alternative measures to reduce the risk of adverse effects are preferable or suffice) requires clinicians to consider individual patient circumstances and also patient preferences. In cases where an adverse drug reaction from antidepressants is suspected (e.g., sexual dysfunction or insomnia), it is also important to carefully consider whether there may be alternative causes prior to changing treatment. In addition, whether and to which extent the implementation of the indicators developed here produces a net benefit to patients and/or health care systems requires evaluation in prospective studies.

## Conclusions

This study has identified a comprehensive set of clinical situations that require a timely critical review of the continuation or deprescribing of antidepressants. It thereby closes an important gap in the current clinical guidelines, which has the potential to counterbalance the use of antidepressants in situations, where they have no relevant benefit, no longer have relevant benefit, or are associated with a high risk of harm. While antidepressants have an irreplaceable role in the treatment of moderate to severe forms of depression and anxiety disorders, in some cases (e.g., in combination with comedication, comorbidity, or age), the risks may outweigh the benefits of therapy, particularly in cases involving milder symptoms as frequently observed in primary care. If the use of the indicators developed here leads to a negative benefit-risk assessment, decisions to deprescribe antidepressant treatment should also take into account the potential harms of deprescribing, including withdrawal symptoms and a potential relapse of symptoms (which may occur with some latency), particularly in those with a history of severe psychiatric disorders. It is also important to note that in some cases, dose reduction or switching to a safer antidepressant may be a better alternative than discontinuation. The explicit indicators of high-risk and overprescribing of antidepressants developed here may be used directly by patients and health care providers in primary care, as well as integrated within clinical decision support tools, in order to improve the overall risk/benefit balance of this commonly prescribed class of prescription drugs. Further research is underway (as part of the POKAL project [45]) to examine the prevalence and longitudinal time trends of the developed indicators using claims data, to examine their acceptability among primary care clinicians, and to evaluate the performance (sensitivity and specificity) of the indicator set in identifying actual opportunities for antidepressant deprescribing.

### Supplementary Information

**Additional file 1**. Search strategy examples.

**Additional file 2.** Expert ratings of round two of the RAM-Survey for high-risk prescribing.

**Additional file 3.** Table with corresponding references and comments in the event of changes between round 2 and round 3 of the RAM-assessment.

**Additional file 4.** Expert ratings of round two of the RAM-Survey for overprescribing.

## Data Availability

The data generated during this study are included in this published article in Tables 3 and 4 [and in Additional files 2 and 4]. Further supporting materials relating to the RAM process described in this article are available upon request.

## Abbreviations

ADR: Adverse drug reaction
FORTA: Fit fOR The Aged
MAOI: Monoamine oxidase inhibitors
NSAID: Nonsteroidal anti-inflammatory drugs
PIM: Potentially inappropriate medication
RAM: RAND/UCLA Appropriateness Method
SNRI: Selective serotonin-norepinephrine reuptake inhibitors
SSRI: Selective serotonin reuptake inhibitors
STOPP/START: Screening Tool of Older Person’s Prescriptions/Screening Tool to Alert doctors to Right Treatment
TCA: Tricyclic antidepressant
