# Supplementary material for: Deprescribing of antidepressants: development of indicators of high-risk and overprescribing using the RAND/UCLA Appropriateness Method
Source: BMC Med. 2024 May 13;22:193. doi: 10.1186/s12916-024-03397-w (PMC11089726; doi:10.1186/s12916-024-03397-w)
Supplement: Supplementary file 1 — Additional file 1. Search strategy examples. [file 12916_2024_3397_MOESM1_ESM.pdf]

DEPRESCRIBING OF ANTIDEPRESSANTS: DEVELOPMENT OF INDICATORS OF HIGH-RISK AND OVERPRESCRIBING USING THE RAND/UCLA APPROPRIATENESS METHOD

Additional file 1: Search strategy examples

eTable 1: Examples for search strategy in PubMed/MEDLINE or EMBASE for ADRs of interest

Search strategy	
Antidepressant Search #1:	(antidepressive agents [MeSH Terms]) OR (Antidepressive Agents, Second Generation [Mesh Terms]) OR (Antidepressive Agents, Tricyclic [Mesh Terms]) OR (Selective serotonin-reuptake inhibitor [MeSH Terms]) OR ("selective serotonin-reuptake inhibitor" [All fields]) OR ("serotonin-norepinephrine reuptake inhibitors" [All fields]) OR („tricyclic antidepressant" [All fields]) OR ("antidepressant") OR ("antidepressants") OR (Bupropion [MeSH Terms]) OR (Trazodone [MeSH Terms]) OR (Mirtazapine [MeSH Terms]) OR (inhibitor, monoamine oxidase [MeSH Terms]) OR (Agomelatine [All fields])
Search terms for adverse drug reaction of interest combined with terms for antidepressants:	
Orthostatic hypotension/dizziness	((Search #1) AND ((hypotension, orthostatic [MeSH Terms]) OR (low blood pressure [MeSH Terms]) OR ("orthostatic hypotension"[All Fields]) OR ("low blood pressure"[All Fields]) OR (hypotension[All Fields]) OR (hypotonia[All Fields])))
Hypertension	((Search #1) AND ((hypertension [MeSH Terms]) OR (blood pressure [MeSH Terms]) OR ("hypertension"[All Fields]) OR ("high blood pressure"[All Fields]) OR („uncontrolled hypertension"[All Fields])))
Falls and fall-related injuries	((Search #1) AND ((„fall-risk"[All Fields]) OR ("fall"[All Fields]) OR („falls"[All Fields]) OR („fall-related injuries"[All Fields]) OR ("fracture"[All Fields]) OR (bone fracture[MeSH Terms])))
Serotonin Syndrome	((Search #1) AND ((Serotonin syndrome[MeSH Terms]) OR ("serotonin syndrome"[All fields]) OR ("serotonin toxicity"[All fields])))
Gastrointestinal bleeding	((Search #1) AND ((gastrointestinal hemorrhage[MeSH Terms]) OR (“gastrointestinal bleeding”[All Fields]) OR (“gastrointestinal hemorrhage”[All fields]) OR (“gastrointestinal haemorrhage”[All fields])))
Bleeding	((Search #1) AND ((intracranial hemorrhages[MeSH Terms]) OR (Hemorrhagic Stroke[MeSH Terms]) OR ("intracranial bleeding"[All fields]) OR ("intracranial hemorrhage"[All fields]) OR ("intracranial haemorrhage"[All fields]) OR (bleeding[All fields]) OR (hemorrhage[All fields]) OR ("major bleeding"[All fields])))
Hyponatremia	((Search #1) AND ((Hyponatremia [Mesh Terms]) OR (Inappropriate ADH Syndrome [Mesh Terms]) OR (hyponatremia[All Fields]) OR (hyponatraemia[All Fields])))
