# Supplementary material for: Deprescribing of antidepressants: development of indicators of high-risk and overprescribing using the RAND/UCLA Appropriateness Method
Source: BMC Med. 2024 May 13;22:193. doi: 10.1186/s12916-024-03397-w (PMC11089726; doi:10.1186/s12916-024-03397-w)
Supplement: Supplementary file 2 — Additional file 2. Expert ratings of round two of the RAM-Survey for high-risk prescribing. [file 12916_2024_3397_MOESM2_ESM.pdf]

DEPRESCRIBING OF ANTIDEPRESSANTS: DEVELOPMENT OF INDICATORS OF HIGH-RISK AND OVERPRESCRIBING USING THE RAND/UCLA APPROPRIATENESS METHOD

Additional file 2: Expert ratings of round two of the RAM-Survey for high-risk prescribing

eTable 2: Expert ratings of round two of the RAM-Survey for high-risk prescribing

<u>Indicators of potential high-risk prescribing</u>

Indicators of potential high-risk prescribing	Necessity to review: Nr. of experts rating: Not necessary (1-3)	Necessity to review: Nr. of experts rating: Might be necessary (4-6)	Necessity to review: Nr. of experts rating: Clearly necessary (7-9)	Necessity to review: Median	**Likelihood of harm Median**	**Severity of harm Median**
**A. ADR Exacerbation of heart failure**						
1. Chronic heart failure and prescribed any antidepressant.	1	6	3	5	5	5
2. Chronic heart failure and prescribed TCA.	0	0	10	8	7	
3. Chronic heart failure and prescribed SNRI.	1	3	6	7	5	
4. Chronic heart failure and prescribed SSRI.	1	6	3	5	4	
5. Chronic heart failure and prescribed TCA in doses < 50 mg/day.	2	6	2	6	3	
6. Chronic heart failure and prescribed TCA in doses ≥ 50 mg/day but < 100mg/day.	1	2	7	7	6	
7. Chronic heart failure and prescribed TCA ≥ 100 mg/day but < 200 mg/day.	0	1	9	8	7	
8. Chronic heart failure and prescribed TCA ≥ 200 mg/day.	0	0	10	9	8	
**A. ADR Exacerbation of coronary heart disease**						
9. Aged < 65 years with coronary heart disease and prescribed TCA.	1	4	5	7	5	6
10. Aged ≥ 65 years with coronary heart disease and prescribed TCA.	0	1	9	8	7	
11. Coronary heart disease and prescribed TCA in doses < 50 mg/day.	2	6	2	6	3	
12. Coronary heart disease and prescribed TCA in doses ≥ 50 mg/day but < 100mg/day.	1	3	6	7	5	
13. Coronary heart disease and prescribed TCA ≥ 100 mg/day but < 200 mg/day.	0	2	8	8	7	
14. Coronary heart disease and prescribed TCA ≥ 200 mg/day.	0	0	10	9	8	
**A. ADR QTc prolongation**						
15. Aged ≥ 65 years and prescribed a single drug with a *known** risk of TdP and that drug is citalopram ≤ 20mg daily or escitalopram ≤ 10mg daily. (*see evidence document)	1	8	1	5	4	7
16. Aged ≥ 65 years and prescribed a single drug with a *known* risk of TdP and that drug is citalopram > 20mg daily or escitalopram > 10mg daily.	0	4	6	7	5	
17. Aged < 65 years and co-prescribed citalopram at a dose of > 20mg or escitalopram at a dose > 10mg and an interacting CYP2C19 Inhibitor (strong or moderate inhibitors according to the Flockhart Table*). (*see evidence document)	1	6	3	6	5	
18. Aged ≥ 65 years and co-prescribed citalopram at a dose of > 20mg or escitalopram at a dose > 10mg and an interacting CYP2C19 Inhibitor (strong or moderate inhibitors according to the Flockhart Table).	0	2	8	7	7	
19. Aged < 65 years and co-prescribed an antidepressant with a *known* risk of TdP and one or more further drugs with a risk of TdP.	0	2	8	8	5	
20. Aged < 65 years and co-prescribed an antidepressant with a *possible** risk of TdP and one or more further drugs with a risk of TdP. (*see evidence document)	2	2	6	7	4	

21. Aged < 65 years and co-prescribed an antidepressant with a conditional* risk of TdP and one or more further drugs with a risk of TdP.	1	4	5	7	4	
22. Aged ≥ 65 years and prescribed a single antidepressant with a *known* risk of TdP.	0	8	2	5	5	
23. Aged ≥ 65 years and co-prescribed an antidepressant with a *known* risk of TdP and one or more further drugs with a risk of TdP.	0	2	8	8	7	
24. Aged ≥ 65 years and co-prescribed an antidepressant with a *possible* risk of TdP and one or more further drugs with a risk of TdP.	0	4	6	7	6	
25. Aged ≥ 65 years and co-prescribed an antidepressant with a conditional risk of TdP and one or more further drugs with a risk of TdP.	1	3	6	7	5	
26. Female, aged ≥ 65 years and prescribed a single antidepressant with a *known* risk of TdP.	0	4	6	7	6	
27. Female, aged ≥ 65 years and co-prescribed an antidepressant with a *known* risk of TdP and one or more further drugs with a risk of TdP.	0	1	9	8	6	
28. Female, aged ≥ 65 years and co-prescribed an antidepressant with a *possible* risk of TdP and one or more further drugs with a risk of TdP.	0	2	8	7	6	
29. Female, aged ≥ 65 years and co-prescribed an antidepressant with a conditional risk of TdP and one or more further drugs with a risk of TdP.	0	2	8	7	6	
30. Chronic heart failure and prescribed a single antidepressant with a *known* risk of TdP.	0	6	4	6	5	
31. Chronic heart failure and co-prescribed an antidepressant with a *known* risk of TdP and one or more further drugs with a risk of TdP.	0	0	10	7	7	
32. Chronic heart failure and co-prescribed an antidepressant with a *possible* risk of TdP and one or more further drugs with a risk of TdP.	0	4	6	7	6	
33. Chronic heart failure and co-prescribed an antidepressant with a *conditional** risk of TdP and one or more further drugs with a risk of TdP. (*see evidence document)	0	5	5	7	5	
34. Cardiac conduction disorders (e.g., congenital long QT-Syndrome) and prescribed a single antidepressant with a *known* risk of TdP.	0	1	9	8	7	
35. Cardiac conduction disorders and co-prescribed an antidepressant with a *known* risk of TdP and one or more further drugs with a risk of TdP.	0	0	10	8	8	
36. Cardiac conduction disorders and co-prescribed an antidepressant with a *possible* risk of TdP and one or more further drugs with a risk of TdP.	0	1	9	7	6	
37. Cardiac conduction disorders and co-prescribed an antidepressant with a *conditional* risk of TdP and one or more further drugs with a risk of TdP.	0	2	8	7	6	
38. History of recurrent hypokalaemia (<3.0 mmol/L) and prescribed a single antidepressant with a *known* risk of TdP.	0	2	8	8	6	
39. History of recurrent hypokalaemia (<3.0 mmol/L) and co-prescribed an antidepressant with a *known* risk of TdP and one or more further drugs with a risk of TdP.	0	0	10	8	7	
40. History of recurrent hypokalaemia (<3.0 mmol/L) and co-prescribed an antidepressant with a *possible* risk of TdP and one or more further drugs with a risk of TdP.	0	0	10	7	6	
41. History of recurrent hypokalaemia (<3.0 mmol/L) and co-prescribed an antidepressant with a *conditional* risk of TdP and one or more further drugs with a risk of TdP.	0	3	7	7	6	
**B. ADR Bradycardia**						
42. Chronic heart failure and co-prescribed an SSRI which is a strong CYP2D6-inhibitor (fluoxetin, paroxetin) and β-blocker (metoprolol, propranolol).	0	0	10	8	8	5
**C. ADR Uncontrolled hypertension**						

43. Neither chronic heart failure nor coronary heart disease but uncontrolled hypertension stage 1 (SBP 140-159, DBP 90-99) and prescribed an antidepressant known to increase blood pressure (SNRI or buproprion or MAOI).	1	6	3	6	4	
44. Neither chronic heart failure nor coronary heart disease but uncontrolled hypertension stage 2 (SBP 160-179, DBP 100-109) and prescribed an antidepressant known to increase blood pressure (SNRI or buproprion or MAOI).	0	3	7	7	5	
45. Neither chronic heart failure nor coronary heart disease but uncontrolled hypertension stage 3 (SBP ≥ 180, DBP ≥ 110) and prescribed an antidepressant known to increase blood pressure (SNRI or buproprion or MAOI).	0	2	8	8	7	
46. Chronic heart failure or coronary heart disease with uncontrolled hypertension stage 1 (SBP 140-159, DBP 90-99) and prescribed an antidepressant known to increase blood pressure (SNRI or buproprion or MAOI).	1	4	5	7	6	
47. Chronic heart failure or coronary heart disease with uncontrolled hypertension stage 2 (SBP 160-179, DBP 100-109) and prescribed an antidepressant known to increase blood pressure (SNRI or buproprion or MAOI).	0	2	8	8	7	
48. Chronic heart failure or coronary heart disease with uncontrolled hypertension stage 3 (SBP ≥ 180, DBP ≥ 110) and prescribed an antidepressant known to increase blood pressure (SNRI or buproprion or MAOI).	0	0	10	9	8	5
49. Chronic heart failure or coronary heart disease without a known history of uncontrolled hypertension prescribed a single antidepressant known to increase blood pressure (SNRI or buproprion or MAOI).	0	7	3	6	5	
50. Chronic heart failure or coronary heart disease without a known history of uncontrolled hypertension co-prescribed an antidepressant known to increase blood pressure (SNRI or buproprion or MAOI) with one further drug known to increase blood pressure.	0	4	6	7	6	
51. Chronic heart failure or coronary heart disease without a known history of uncontrolled hypertension co-prescribed an antidepressant known to increase blood pressure (SNRI or buproprion or MAOI) with two further drugs known to increase blood pressure.	0	2	8	8	7	
**D. ADR Tachycardia**						
52. Neither chronic heart failure nor coronary heart disease but prescribed a TCA and has developed uncontrolled tachycardia.	0	1	9	7	6	
53. Neither chronic heart failure nor coronary heart disease but prescribed a SNRI and has developed uncontrolled tachycardia.	0	3	7	7	6	
54. Neither chronic heart failure nor coronary heart disease but prescribed a MAOI and has developed uncontrolled tachycardia.	0	1	9	8	6	
55. Chronic heart failure or coronary heart disease, prescribed a TCA and has developed uncontrolled tachycardia.	0	0	10	9	7	6
56. Chronic heart failure or coronary heart disease, prescribed a SNRI and has developed uncontrolled tachycardia.	0	2	8	8	7	
57. Chronic heart failure or coronary heart disease, prescribed a MAOI and has developed uncontrolled tachycardia.	0	1	8	9	7	
**E. ADR Gastrointestinal bleeding**						
58. Aged < 65 years and co-prescribed SSRI and a single of the following drugs known to increase the risk of gastrointestinal bleeding: antiplatelet or anticoagulant or NSAID (without gastrointestinal protection).	3	3	4	6	5	
59. Aged < 65 years and co-prescribed SSRI and a single of the following drugs known to increase the risk of gastrointestinal bleeding: antiplatelet or anticoagulant or NSAID (with gastrointestinal protection).	4	6	0	4	4	
60. Aged < 65 years and co-prescribed SSRI and two or more of the following drugs: antiplatelet and/or anticoagulant and/or NSAID (without gastrointestinal protection).	0	6	4	6	6	
61. Aged < 65 years and co-prescribed SSRI and two or more of the following drugs: antiplatelet and/or anticoagulant and/or NSAID (with gastrointestinal protection).	2	5	3	6	5	6
62. Aged ≥ 65 years and co-prescribed SSRI and a single of the following drugs: antiplatelet or anticoagulant or NSAID (without gastrointestinal protection).	0	3	7	8	6	
63. Aged ≥ 65 years and co-prescribed SSRI and a single of the following drugs: antiplatelet or anticoagulant or NSAID (with gastrointestinal protection).	2	4	4	6	4	

64. Aged ≥ 65 years and co-prescribed SSRI and two or more of the following drugs: antiplatelet and/or anticoagulant and/or NSAID (without gastrointestinal protection).	0	2	8	9	7
65. Aged ≥ 65 years and co-prescribed SSRI and two or more of the following drugs: antiplatelet and/or anticoagulant and/or NSAID (with gastrointestinal protection).	1	3	6	7	5
66. At least one risk factor (history of peptic ulcer disease, gastrointestinal bleeding or haemophilia) and prescribed SSRI (without gastrointestinal protection).	1	6	3	5	5
67. At least one risk factor (history of peptic ulcer disease, gastrointestinal bleeding or haemophilia) and prescribed SSRI (with gastrointestinal protection).	2	6	2	6	5
68. At least one risk factor (history of peptic ulcer disease, gastrointestinal bleeding or haemophilia) and co-prescribed SSRI and a single of the following drugs: antiplatelet or anticoagulant or NSAID (without gastrointestinal protection).	0	4	6	7	6
69. At least one risk factor (history of peptic ulcer disease, gastrointestinal bleeding or haemophilia) and co-prescribed SSRI and a single of the following drugs: antiplatelet or anticoagulant or NSAID (with gastrointestinal protection).	2	2	6	7	6
70. At least one risk factor (history of peptic ulcer disease, gastrointestinal bleeding or haemophilia) and co-prescribed SSRI and two or more of the following drugs: antiplatelet and/or anticoagulant and/or NSAID (without gastrointestinal protection).	0	2	8	9	7
71. At least one risk factor (history of peptic ulcer disease, gastrointestinal bleeding or haemophilia) and co-prescribed SSRI and two or more of the following drugs: antiplatelet and/or anticoagulant and/or NSAID (with gastrointestinal protection).	1	3	6	8	6
72. At least one risk factor (history of peptic ulcer disease, gastrointestinal bleeding, haemophilia or aged ≥ 65 years) and prescribed SNRI or TCA (without gastrointestinal protection).	1	3	6	7	6
73. At least one risk factor (history of peptic ulcer disease, gastrointestinal bleeding, haemophilia or aged ≥ 65 years) and prescribed SNRI or TCA (with gastrointestinal protection).	2	3	5	7	5
74. At least one risk factor (history of peptic ulcer disease, gastrointestinal bleeding, haemophilia or aged ≥ 65 years) and co-prescribed SNRI or TCA and a single of the following drugs: antiplatelet or anticoagulant or NSAID (without gastrointestinal protection).	1	2	7	8	6
75. At least one risk factor (history of peptic ulcer disease, gastrointestinal bleeding, haemophilia or aged ≥ 65 years) and co-prescribed SNRI or TCA and a single of the following drugs: antiplatelet or anticoagulant or NSAID (with gastrointestinal protection).	2	3	5	7	5
76. At least one risk factor (history of peptic ulcer disease, gastrointestinal bleeding, haemophilia or aged ≥ 65 years) and co-prescribed SNRI or TCA and two or more of the following drugs: antiplatelet or anticoagulant or NSAID (without gastrointestinal protection).	1	1	8	9	8
77. NAt least one risk factor (history of peptic ulcer disease, gastrointestinal bleeding, haemophilia or aged ≥ 65 years) and co-prescribed SNRI or TCA and two or more of the following drugs: antiplatelet or anticoagulant or NSAID (with gastrointestinal protection).	2	1	7	8	7
**F. ADR Bleeding**					
78. History of bleeding event and co-prescribed SSRI and a single of the following drugs: anticoagulant or antiplatelet.	0	5	5	7	5
79. History of bleeding event and co-prescribed SSRI and two or more of the following drugs: anticoagulant and antiplatelet.	0	2	8	8	7
80. Stroke and co-prescribed SSRI and a single of the following drugs: anticoagulant or antiplatelet.	0	6	4	6	4
81. Stroke and co-prescribed SSRI and two or more of the following drugs: anticoagulant and antiplatelet.	0	4	6	7	6
82. Dementia and co-prescribed SSRI and a single of the following drugs: anticoagulant or antiplatelet.	0	6	4	5	4
83. Dementia and co-prescribed SSRI and two or more of the following drugs: anticoagulant and antiplatelet.	0	5	5	7	6
84. Aged ≥ 65 years without a known dementia and co-prescribed SSRI and a single of the following drugs: anticoagulant or antiplatelet.	0	6	4	6	5

85. Aged ≥ 65 years without a known dementia and co-prescribed SSRI and two or more of the following drugs: anticoagulant and antiplatelet.	0	3	7	7	6	
**G. ADR Constipation**						
86. Prescribed TCA < 100 mg/day and patient has persistent constipation.	1	7	2	5	5	4
87. Prescribed TCA ≥ 100 mg/day but < 200 mg/day and patient has persistent constipation.	0	7	3	6	5	
88. Prescribed TCA ≥ 200 mg/day and patient has persistent constipation.	0	2	8	7	6	
89. Prescribed a TCA without further drugs known to have constipating effects*, and patient has persistent constipation. (*see evidence document)	1	2	7	7	6	
90. Prescribed a TCA and one further drug known to have constipating effects, and patient has persistent constipation.	0	1	9	7	6	
91. Prescribed a TCA and two further drugs known to have constipating effects, and patient has persistent constipation.	0	1	9	8	7	
92. Aged ≥ 65 years without a known history of persistent constipation, prescribed a TCA and one further non-anticholinergic drug (e.g. opioids, verapamil) known to have constipating effects.	1	5	4	6	5	
93. Aged ≥ 65 years without a known history of persistent constipation, prescribed a TCA and two further non-anticholinergic drugs (e.g. opioids, verapamil) known to have constipating effects.	0	3	7	7	6	
94. Prescribed a SNRI without further drugs known to have constipating effects, and patient has persistent constipation.	1	8	1	5	4	
95. Prescribed a SNRI and one further drug known to have constipating effects, and patient has persistent constipation.	1	8	1	5	5	
96. Prescribed a SNRI and two further drugs known to have constipating effects, and patient has persistent constipation.	1	6	3	6	6	
**H. ADR Cognitive impairment/decline**						
97. Cognitive impairment and prescribed TCA in doses < 50 mg/day.	1	7	2	6	4	6
98. Cognitive impairment and prescribed TCA in doses ≥ 50 mg/day but < 100 mg/day.	1	2	7	7	6	
99. Cognitive impairment and prescribed TCA ≥ 100 mg/day but < 200 mg/day.	0	1	9	8	6	
100. Cognitive impairment and prescribed TCA ≥ 200 mg/day.	0	1	9	9	7	
101. Cognitive impairment, prescribed a TCA and no further anticholinergic drugs, and the total anticholinergic burden is 3.	0	2	8	8	5	
102. Cognitive impairment, prescribed a TCA with further drugs, and the total anticholinergic burden is 4	0	2	8	8	6	
103. Cognitive impairment, prescribed a TCA with further drugs, and the total anticholinergic burden is ≥ 5.	0	1	9	9	8	
104. Cognitive impairment, prescribed paroxetine or opipramol and no further anticholinergic drugs, and the total anticholinergic burden is 2.	1	8	1	4	3	
105. Cognitive impairment, prescribed paroxetine or opipramol with further drugs, and the total anticholinergic burden is 3.	0	7	3	5	4	
106. Cognitive impairment, prescribed paroxetine or opipramol with further drugs, and the total anticholinergic burden is 4.	0	6	4	6	5	
107. Cognitive impairment, prescribed paroxetine or opipramol with further drugs, and the total anticholinergic burden is ≥ 5.	0	1	9	8	6	
108. Cognitive impairment and prescribed 1 DDD citalopram daily.	10	0	0	3	2	
109. Cognitive impairment and prescribed ≥ 1,5 DDD citalopram daily.	3	7	0	4	3	
110. Cognitive impairment and prescribed 1 DDD SSRI other than citalopram and paroxetine.	8	2	0	3	2	
111. Cognitive impairment and prescribed ≥ 1,5 DDD SSRI other than citalopram and paroxetine.	3	6	1	4	3	
112. Cognitive impairment and prescribed 1 DDD mirtazapine daily.	8	2	0	3	2	
113. Cognitive impairment and prescribed ≥ 1,5 DDD mirtazapine daily.	4	6	0	4	3	
114. Cognitive impairment and prescribed trazodone.	4	5	1	4	4	
115. Cognitive impairment and prescribed SNRI (venlafaxine, duloxetine).	4	5	1	4	3	
**I. ADR Delirium**						

116. At least one risk factor (cognitive impairment, dementia or history of delirium) and prescribed TCA and no further drugs known to induce delirium.	0	9	1	5	4	
117. At least one risk factor (cognitive impairment, dementia or history of delirium) and prescribed TCA and one further non-anticholinergic drug known to induce delirium (e.g. benzodiazepines, opioids).	0	5	5	7	5	
118. At least one risk factor (cognitive impairment, dementia or history of delirium) and prescribed TCA and two further non-anticholinergic drugs known to induce delirium (e.g. benzodiazepines, opioids).	0	2	8	8	6	
119. Aged ≥ 65 years and prescribed TCA and one further non-anticholinergic drug known to induce delirium (e.g. benzodiazepines, opioids).	0	6	4	6	5	7
120. Aged ≥ 65 years and prescribed TCA and two further non-anticholinergic drugs known to induce delirium (e.g. benzodiazepines, opioids).	0	3	7	8	6	
121. At least one risk factor (cognitive impairment, dementia or history of delirium) and prescribed TCA with further anticholinergic drugs, and the total anticholinergic burden is 4.	0	3	7	8	6	
122. At least one risk factor (cognitive impairment, dementia or history of delirium) and prescribed TCA with further anticholinergic drugs, and the total anticholinergic burden is ≥ 5.	0	1	9	9	7	
**J. ADR Serotonin syndrome**						
123. Co-prescribed SSRI/SNRI/TCA (clomipramine, imipramine) and MAOI.	0	3	7	9	7	
124. Co-prescribed SSRI with one further serotonergic drug* other than MAOI. (*see evidence document)	1	5	4	6	4	
125. Co-prescribed SSRI with two further serotonergic drugs other than MAOI.	0	3	7	7	5	
126. Co-prescribed SSRI with three further serotonergic drugs other than MAOI.	0	1	9	8	6	
127. Co-prescribed SNRI with one further serotonergic drug other than MAOI.	1	6	3	6	4	
128. Co-prescribed SNRI with two further serotonergic drugs other than MAOI.	0	6	4	6	5	
129. Co-prescribed SNRI with three further serotonergic drugs other than MAOI.	0	1	9	8	6	7
130. Co-prescribed MAOI with one further serotonergic drug other than SSRI/SNRI/TCA (clomipramine, imipramine).	0	3	7	8	6	
131. Co-prescribed MAOI with two further serotonergic drugs other than SSRI/SNRI/TCA (clomipramine, imipramine).	0	2	8	9	7	
132. Co-prescribed MAOI with three further serotonergic drugs other than SSRI/SNRI/TCA (clomipramine, imipramine).	0	0	10	9	8	
133. Co-prescribed a TCA (clomipramine/imipramine) with one further serotonergic drug other than MAOI.	0	8	2	6	5	
134. Co-prescribed a TCA (clomipramine/imipramine) with two further serotonergic drugs other than MAOI.	0	6	4	6	5	
135. Co-prescribed a TCA (clomipramine/imipramine) with three further serotonergic drugs other than MAOI.	0	3	7	7	6	
**K. ADR Stroke**						
136. Patient with a history of stroke is prescribed an antidepressant other than SSRI or TCA.	4	6	0	4	3	
137. Patient with a history of stroke is prescribed SSRI.	6	3	1	3	3	
138. Patient with a history of stroke is prescribed TCA.	1	5	4	6	5	8
139. Aged < 65 years at high risk of cardiovascular events is prescribed SSRI.	2	8	0	4	4	
140. Aged ≥ 65 years at high risk of cardiovascular events is prescribed SSRI.	2	8	0	5	5	
**L. ADR Falls and fall-related injuries**						
141. Aged ≥ 65 years and prescribed one single antidepressant with sedating, anticholinergic or orthostatic properties (TCA or mirtazapine or trazodone).	0	7	3	5	5	7
142. Aged ≥ 65 years and co-prescribed an antidepressant with sedating, anticholinergic or orthostatic properties (TCA or mirtazapine or trazodone) with one further fall-risk increasing drug*. (*see evidence document)	0	3	7	7	6	

143. Aged ≥ 65 years and co-prescribed an antidepressant with sedating, anticholinergic or orthostatic properties (TCA or mirtazapine or trazodone) with two or more further fall-risk increasing drugs.	0	1	9	8	7
144. Aged ≥ 65 years and prescribed one single antidepressant with activating properties (SSRI or SNRI).	2	8	0	4	4
145. Aged ≥ 65 years and co-prescribed an antidepressant with activating properties (SSRI or SNRI) with one further fall-risk increasing drug.	1	7	2	6	5
146. Aged ≥ 65 years and co-prescribed an antidepressant with activating properties (SSRI or SNRI) with two or more further fall-risk increasing drugs.	1	1	8	8	7
147. History of fall and prescribed one single antidepressant with sedating, anticholinergic or orthostatic properties (TCA or mirtazapine or trazodone).	0	4	6	7	5
148. History of fall and co-prescribed an antidepressant with sedating, anticholinergic or orthostatic properties (TCA or mirtazapine or trazodone) with one further fall-risk increasing drug.	0	1	9	8	7
149. History of fall and co-prescribed an antidepressant with sedating, anticholinergic or orthostatic properties (TCA or mirtazapine or trazodone) with two or more further fall-risk increasing drugs.	0	1	9	9	7
150. History of fall and prescribed one single antidepressant with activating properties (SSRI or SNRI).	2	5	3	6	4
151. History of fall and co-prescribed an antidepressant with activating properties (SSRI or SNRI) with one further fall-risk increasing drug.	1	3	6	7	5
152. History of fall and co-prescribed an antidepressant with activating properties (SSRI or SNRI) with two or more further fall-risk increasing drugs.	1	2	7	8	6
153. Cognitive impairment and prescribed one single antidepressant with sedating, anticholinergic or orthostatic properties (TCA or mirtazapine or trazodone).	0	2	8	8	6
154. Cognitive impairment and co-prescribed an antidepressant with sedating, anticholinergic or orthostatic properties (TCA or mirtazapine or trazodone) with one further fall-risk increasing drug.	0	2	8	8	7
155. Cognitive impairment and co-prescribed an antidepressant with sedating, anticholinergic or orthostatic properties (TCA or mirtazapine or trazodone) with two or more further fall-risk increasing drugs.	0	1	9	9	8
156. Cognitive impairment and prescribed one single antidepressant with activating properties (SSRI or SNRI).	3	6	1	6	3
157. Cognitive impairment and co-prescribed an antidepressant with activating properties (SSRI or SNRI) with one further fall-risk increasing drug.	1	2	7	7	5
158. Cognitive impairment and co-prescribed an antidepressant with activating properties (SSRI or SNRI) with two or more further fall-risk increasing drugs.	1	2	7	8	6
159. At least one risk factor for falls (aged ≥ 65 years, history of fall or cognitive impairment) and co-prescribed SSRI and benzodiazepine/Z-hypnotic.	1	0	9	8	7
160. At least one risk factor for falls (aged ≥ 65 years, history of fall or cognitive impairment) and co-prescribed SSRI and benzodiazepin/Z-hypnotic ≤ 4 weeks.	1	0	9	8	7
161. At least one risk factor for falls (aged ≥ 65 years, history of fall or cognitive impairment) and co-prescribed SSRI and benzodiazepin/Z-hypnotic > 4 weeks.	1	0	9	9	7
162. Known osteoporosis and prescribed SSRI.	2	6	2	6	4
163. History of low impact fracture and prescribed SSRI.	0	7	3	6	4
164. Aged ≥ 65 years without known osteoporosis and prescribed SSRI.	5	5	0	4	3
165. Aged ≥ 75 years without known osteoporosis and prescribed SSRI.	2	7	1	5	6

M. ADR Orthostatic hypotension/dizziness

166. Aged < 65 years, prescribed an antidepressant with an increased risk of orthostatic hypotension (TCA or trazodone or MAOI) and has developed persistent orthostatic hypotension/dizziness.	1	1	8	7	5	
167. Aged ≥ 65 years, prescribed an antidepressant with an increased risk of orthostatic hypotension (TCA or trazodone or MAOI) and has developed persistent orthostatic hypotension/dizziness.	0	1	9	9	7	
168. Aged ≥ 65 years without a known orthostatic hypotension/dizziness, prescribed an antidepressant with an increased risk of orthostatic hypotension (TCA or trazodone or MAOI).	1	9	0	6	5	
169. Aged ≥ 65 years without a known orthostatic hypotension/dizziness, co-prescribed an antidepressant (TCA or trazodone or MAOI) with one further drug with known blood pressure lowering effect*. (*see evidence document)	1	2	7	7	5	
170. Aged ≥ 65 years without a known orthostatic hypotension/dizziness, co-prescribed an antidepressant (TCA or trazodone or MAOI) with two or more further drugs with known blood pressure lowering effect.	1	0	9	8	6	6
171. Aged < 65 years, prescribed an antidepressant with a lower risk of orthostatic hypotension (SSRI or SNRI or mirtazapine) and has developed persistent orthostatic hypotension/dizziness.	0	6	4	6	4	
172. Aged ≥ 65 years, prescribed an antidepressant with a lower risk of orthostatic hypotension (SSRI or SNRI or mirtazapine) and has developed persistent orthostatic hypotension/dizziness.	0	2	8	7	6	
173. Aged ≥ 65 years without a known orthostatic hypotension/dizziness, prescribed an antidepressant with a lower risk of orthostatic hypotension (SSRI or SNRI or mirtazapine).	7	3	0	3	3	
174. Aged ≥ 65 years without a known orthostatic hypotension/dizziness, co-prescribed an antidepressant (SSRI or SNRI or mirtazapine) with one further drug with known blood pressure lowering effect.	2	6	2	6	5	
175. Aged ≥ 65 years without a known orthostatic hypotension/dizziness, co-prescribed an antidepressant (SSRI or SNRI or mirtazapine) with two or more further drugs with known blood pressure lowering effect.	2	0	8	7	6	
**N. ADR Hyponatremia**						
176. Prescribed an antidepressant with an increased risk of hyponatremia (SSRI or SNRI) with current or recent hyponatremia (130-134 mmol/l).	0	6	4	6	6	
177. Prescribed an antidepressant with a moderate or low risk of hyponatremia (TCA or mirtazapine or others) with current or recent hyponatremia (130-134 mmol/l).	2	7	1	5	4	
178. Prescribed an antidepressant with an increased risk of hyponatremia (SSRI or SNRI) with current or recent hyponatremia (<130 mmol/l).	0	3	7	7	7	
179. Prescribed an antidepressant with a moderate or low risk of hyponatremia (TCA or mirtazapine or others) with current or recent hyponatremia (<130 mmol/l).	2	2	6	7	5	
180. Aged ≥ 65 years without a known history of hyponatremia, prescribed an antidepressant with an increased risk of hyponatremia (SSRI or SNRI).	1	9	0	5	4	6
181. Aged ≥ 65 years without a known history of hyponatremia, co-prescribed an antidepressant with an increased risk of hyponatremia (SSRI or SNRI) with one further drug known to cause hyponatremia*. (*see evidence document).	1	6	3	6	5	
182. Aged ≥ 65 years without a known history of hyponatremia, co-prescribed an antidepressant with an increased risk of hyponatremia (SSRI or SNRI) with two or more further drugs known to cause hyponatremia.	1	2	7	7	6	
183. Aged ≥ 65 years without a known history of hyponatremia, prescribed an antidepressant with a moderate or low risk of hyponatremia (TCA or mirtazapine or others).	3	7	0	4	3	
184. Aged ≥ 65 years without a known history of hyponatremia, co-prescribed an antidepressant with a moderate or low risk of hyponatremia (TCA or mirtazapine or others) with one further drug known to cause hyponatremia.	1	7	2	5	4	

185. Aged ≥ 65 years without a known history of hyponatremia, co-prescribed an antidepressant with a moderate or low risk of hyponatremia (TCA or mirtazapine or others) with two or more further drugs known to cause hyponatremia.	1	5	4	6	5	
**O. ADR Hepatic Injury**						
186. Prescribed an antidepressant other than agomelatine and has developed elevated serum transaminase levels (> 3 times the upper normal range).	0	6	4	6	3	6
187. Prescribed agomelatine and has developed elevated serum transaminase levels (> 3 times the upper normal range).	0	0	10	8	5	
188. Hepatic impairment and prescribed agomelatine.	0	0	10	9	7	
**P. ADR Metabolic disturbances/weight gain/weight loss**						
189. Diabetes mellitus and prescribed TCA.	2	5	3	5	5	5
190. Diabetes mellitus and prescribed mirtazapine.	2	4	4	5	4	
191. Prescribed TCA and has BMI 25 to < 30 kg/m².	3	6	1	5	4	
192. Prescribed mirtazapine and has BMI 25 to < 30 kg/m².	2	6	2	5	4	
193. Prescribed TCA and has BMI ≥ 30 kg/m².	2	5	3	5	5	
194. Prescribed mirtazapine and has BMI ≥30 kg/m².	1	6	3	6	5	
195. Prescribed buproprion and has BMI < 18,5 kg/m².	2	7	1	5	4	
**Q. ADR Hypoglycemia**						
196. Prescribed SSRI and has recurrent hypoglycemia.	2	2	6	7	4	6
**R. ADR Voiding disorders**						
197. History of voiding disorders and prescribed TCA < 100 mg/day.	0	7	3	6	4	5
198. History of voiding disorders and prescribed TCA ≥ 100 mg/day, but < 200 mg/day.	0	3	7	7	5	
199. History of voiding disorders and prescribed TCA ≥ 200 mg/day.	0	0	10	8	6	
200. History of voiding disorders, prescribed TCA and no further anticholinergic drugs, and the total anticholinergic burden is 3.	0	5	5	7	5	
201. History of voiding disorders, prescribed TCA with further drugs, and the total anticholinergic burden is 4.	0	2	8	7	6	
202. History of voiding disorders, prescribed TCA with further drugs, and the total anticholinergic burden is ≥ 5.	0	1	9	8	7	
203. History of voiding disorders, prescribed paroxetine or opipramol and no further anticholinergic drugs, and the total anticholinergic burden is 2.	0	10	0	5	4	
204. History of voiding disorders, prescribed paroxetine or opipramol with further drugs, and the total anticholinergic burden is 3.	0	6	4	6	5	
205. History of voiding disorders, prescribed paroxetine or opipramol with further drugs, and the total anticholinergic burden is 4.	0	1	9	8	6	
206. History of voiding disorders, prescribed paroxetine or opipramol with further drugs, and the total anticholinergic burden is ≥ 5.	0	0	10	9	6	
207. History of voiding disorders, prescribed SNRI or NRI.	1	5	4	6	4	
**S. ADR Glaucoma**						
208. Angle closure glaucoma and prescribed TCA < 100 mg/day.	4	4	2	5	3	6
209. Angle closure glaucoma and prescribed TCA ≥ 100 mg/day but < 200 mg/day.	1	6	3	6	5	
210. Angle closure glaucoma and prescribed TCA ≥ 200 mg/day.	0	6	4	6	6	
211. Angle closure glaucoma, prescribed TCA and no further anticholinergic drugs, and the total anticholinergic burden is 3.	1	6	3	5	4	
212. Angle closure glaucoma, prescribed TCA with further drugs, and the total anticholinergic burden is 4.	0	7	3	6	6	
213. Angle closure glaucoma, prescribed TCA with further drugs, and the total anticholinergic burden is ≥ 5.	0	4	6	7	6	
214. Angle closure glaucoma, prescribed paroxetine or opipramol and no further anticholinergic drugs, and the total anticholinergic burden is 2.	4	6	0	4	3	

215. Angle closure glaucoma, prescribed paroxetine or opipramol with further drugs, and the total anticholinergic burden is 3.	0	8	2	6	5	
216. Angle closure glaucoma, prescribed paroxetine or opipramol with further drugs, and the total anticholinergic burden is 4.	0	7	3	6	5	
217. Angle closure glaucoma, prescribed paroxetine or opipramol with further drugs, and the total anticholinergic burden is ≥ 5.	0	2	8	7	6	
218. Patient with an increased risk of angle closure glaucoma and prescribed SNRI.	4	6	0	4	3	
219. Patient with an increased risk of angle closure glaucoma and prescribed SSRI other than paroxetine.	7	2	1	3	2	
**T. ADR Sleep disturbances**						
220. Prescribed an antidepressant with activating properties (SSRI or SNRI or MAOI or buproprion) and has persistent sleeping disturbances.	0	0	10	7	5	6
221. Prescribed an antidepressant with non-activating properties (TCA or mirtazapine or trazodone) and has persistent sleeping disturbances.	2	6	2	5	4	
**U. ADR Sexual dysfunction**						
222. Prescribed SSRI or SNRI and has developed sexual dysfunction.	1	1	8	8	6	5
