# Supplementary material for: Deprescribing of antidepressants: development of indicators of high-risk and overprescribing using the RAND/UCLA Appropriateness Method
Source: BMC Med. 2024 May 13;22:193. doi: 10.1186/s12916-024-03397-w (PMC11089726; doi:10.1186/s12916-024-03397-w)
Supplement: Supplementary file 3 — Additional file 3. Table with corresponding references and comments in the event of changes between round 2 and round 3 of the RAM-assessment. [file 12916_2024_3397_MOESM3_ESM.pdf]

DEPRESCRIBING OF ANTIDEPRESSANTS: DEVELOPMENT OF INDICATORS OF HIGH-RISK AND OVERPRESCRIBING USING THE RAND/UCLA APPROPRIATENESS METHOD

Additional file 3: Corresponding references and comments in the event of changes between round 2 and round 3 of the RAM-assessment

eTable 3: Corresponding references and comments in the event of changes between round 2 and round 3 of the RAM-assessment

High-risk prescribing indicators

RAM-Survey: Third round results	References	Comments
A. Risk of cardiovascular adverse effects		
1. Prescribed SNRI or TCA (in doses ≥ 50 mg/day)[1] or tranylcypromine[2] - and patient has a history of chronic heart failure.<br><br>[1]It cannot be excluded that low-dose TCAs also have significant adverse effects, as evidence on the safety of low-dose TCAs is sparse.<br>[2]Especially when co-administered with tyramine-containing food.	• Bundesärztekammer (BÄK), Kassenärztliche Bundesvereinigung (KBV), Arbeitsgemeinschaft der Wissenschaftlichen Medizinischen Fachgesellschaften (AWMF). Nationale VersorgungsLeitlinie Chronische Herzinsuffizienz –Langfassung, 3. Auflage. Version 3. 2019 [cited: 2023-05-05].<br>• Ladwig KH, Baghai TC, Doyle F, Hamer M, Herrmann-Lingen C, Kunschitz E, Lemogne C, Beresnevaite M, Compare A, von Känel R, Sager HB, Kop WJ. Mental health-related risk factors and interventions in patients with heart failure: a position paper endorsed by the European Association of Preventive Cardiology (EAPC). Eur J Prev Cardiol. 2022 May 25;29(7):1124-1141.	• **TCA (in doses ≥ 50 mg/day) -> this applies to ALL indicators that list TCA:** There is limited evidence on the safety of low-dose TCAs (<50 mg/day). Therefore, it was unanimously agreed by the expert panel that a review of their use, particularly at doses ≥50 mg/day, is necessary.
2. Prescribed TCA (in doses ≥ 50 mg/day)[1] - and patient has a history of ischemic heart disease.	• DGPPN, BÄK, KBV, AWMF (Hrsg.) für die Leitliniengruppe Unipolare Depression*. S3-Leitlinie/Nationale VersorgungsLeitlinie Unipolare Depression – Langfassung, 2. Auflage. Version 5. 2015 [cited: 2023-05-05].	
3. Prescribed >20mg citalopram or >10mg escitalopram daily - and patient is aged ≥65 years (risk of QTc prolongation).	• Lundbeck GmbH. Rote-Hand-Brief vom 31.10.2011 zu Cipramil® (Citalopram): Zusammenhang von Cipramil® (Citalopramhydrobromid/Citalopramhydrochlorid) mit dosisabhängiger QT-Intervall-Verlängerung. 2011 http://www.akdae.de/Arzneimittelsicherheit/RHB/Archiv/2011/20111031.pdf.<br>• Lundbeck GmbH. Rote-Hand-Brief vom 05.12.2011 zu Escitalopram (Cipralex®): Zusammenhang von Escitalopram (Cipralex®) mit dosisabhängiger QT-Intervall-Verlängerung. 2011 http://www.akdae.de/Arzneimittelsicherheit/RHB/Archiv/2011/20111205.pdf.	

4. Prescribed citalopram, escitalopram - and patient has long QT-Syndrome or is at risk of long QT-syndrome (e.g., (advanced) chronic heart failure, ischemic heart disease, myocardial hypertrophy, bradyarrhythmias or an ongoing risk of hypokalaemia[3]).<br>[3]Ongoing risk of hypokalemia means recurrent hypokalemia despite efforts to improve or without prescription of potassium supplements.<br>5. Prescribed citalopram, escitalopram, TCA (in doses ≥ 50 mg/day)[1] - and patient is co-prescribed ≥1 further drug with any risk of TdP[4].<br>[4]Drugs with any risk of TdP (known, possible or conditional) according to: Woosley RL et.al.	• Woosley RL, Heise CW , Gallo T, Tate J, Woosley D and Romero KA, www.CredibleMeds.org, QTdrugs List, [25.05.2022], AZCERT, Inc. 1457 E. Desert Garden Dr., Tucson, AZ 85718.<br>• Sauer AJ, Newton-Cheh C. Clinical and genetic determinants of torsade de pointes risk. Circulation. 2012 Apr 3;125(13):1684-94.<br>• Haverkapm W., Haverkamp F., Medikamentenbedingte QT-Verlängerung und Torsade de pointes: Ein multidisziplinäres Problem**;** Dtsch Arztebl 2002; 99: A 1972–1979 [Heft 28–29]**.**<br>• Bundesärztekammer (BÄK), Kassenärztliche Bundesvereinigung (KBV), Arbeitsgemeinschaft der Wissenschaftlichen Medizinischen Fachgesellschaften (AWMF). Nationale VersorgungsLeitlinie Chronische Herzinsuffizienz –Langfassung, 3. Auflage. Version 3. 2019 [cited: 2023-05-05].	• The classification of antidepressants with TdP risk was based on the CredibleMeds program, developed by the Arizona Center for Education and Research on Therapeutics.
6. Prescribed TCA (in doses ≥ 50 mg/day)[1] or SNRI or bupropion or tranylcypromine[2] - and patient has developed tachycardia.	• Calvi A, Fischetti I, Verzicco I, Belvederi Murri M, Zanetidou S, Volpi R, et al. Antidepressant Drugs Effects on Blood Pressure. Frontiers in cardiovascular medicine. 2021;8:704281.<br>• Teply RM, Packard KA, White ND, Hilleman DE, DiNicolantonio JJ. Treatment of Depression in Patients with Concomitant Cardiac Disease. Progress in Cardiovascular Diseases. 2016;58(5):514-28.	• Expert panel agreed on the necessity of a review in case of tachycardia, regardless if chronic heart failure or coronary heart disease are present or not.
7. Prescribed fluoxetine, paroxetine or bupropion - and patient is co-prescribed metoprolol or propranolol (risk of bradycardia).	• Teply RM, Packard KA, White ND, Hilleman DE, DiNicolantonio JJ. Treatment of Depression in Patients with Concomitant Cardiac Disease. Progress in Cardiovascular Diseases. 2016;58(5):514-28.<br>• Bahar MA, Kamp J, Borgsteede SD, Hak E, Wilffert B. The impact of CYP2D6 mediated drug–drug interaction: a systematic review on a combination of metoprolol and paroxetine/fluoxetine. 2018;84(12):2704-15.<br>• Shin J, Hills NK, Finley PR. Combining Antidepressants with β-Blockers: Evidence of a Clinically Significant CYP2D6 Drug Interaction. Pharmacotherapy. 2020;40(6):507-16.<br>• McCollum DL, Greene JL, McGuire DK. Severe sinus bradycardia after initiation of bupropion therapy: a probable drug-drug interaction with metoprolol. Cardiovasc Drugs Ther. 2004;18(4):329-330.<br>• Flockhart DA, Thacker, D., McDonald, C., Desta, Z. The Flockhart Cytochrome P450 Drug-Drug Interaction Table. Division of Clinical Pharmacology, Indiana University School of Medicine (Updated	• Paroxetine, fluoxetine, and bupropion are potent inhibitors of the CYP2D6 enzyme (according to Flockhart table), and cases of bradycardia (and heart block) have been reported when combined with metoprolol and propranolol, which are substrates of CYP2D6.

	2021). https://drug-interactions.medicine.iu.edu. Accessed [2023-08.28].	
8. Prescribed SNRI or TCA (in doses ≥ 50 mg/day)[1] or bupropion or tranylcypromine[2]<br>- and patient has uncontrolled hypertension.[5]<br><br>9. Prescribed SNRI or TCA (in doses ≥ 50 mg/day)[1] or bupropion or tranylcypromine[2]<br>- and achieving hypertension control requires ≥3 antihypertensive drugs.<br><br>[5](i.e. SBP>140 or SBP>160 depending on the presence or absence of chronic heart failure or coronary heart disease, respectively).	• Whelton PK, Carey RM, Aronow WS, Casey DE, Collins KJ, Himmelfarb CD, et al.: 2017 ACC/AHA/AAPA/ABC/ACPM/AGS/APhA/ASH/ASPC/NMA/PCNA Guideline for the Prevention, Detection, Evaluation, and Management of High Blood Pressure in Adults: A Report of the American College of Cardiology/American Heart Association Task Force on Clinical Practice Guidelines. Hypertension. 2018;71(6):e13-e115.<br>• Calvi A, Fischetti I, Verzicco I, Belvederi Murri M, Zanetidou S, Volpi R, et al. Antidepressant Drugs Effects on Blood Pressure. Front Cardiovasc Med. 2021;8:704281.<br>• Zhong Z, Wang L, Wen X, Liu Y, Fan Y, Liu Z. A meta-analysis of effects of selective serotonin reuptake inhibitors on blood pressure in depression treatment: outcomes from placebo and serotonin and noradrenaline reuptake inhibitor controlled trials. Neuropsychiatr Dis Treat. 2017 Nov 7;13:2781-2796.<br>• Teply RM, Packard KA, White ND, Hilleman DE, DiNicolantonio JJ. Treatment of Depression in Patients with Concomitant Cardiac Disease. Progress in Cardiovascular Diseases. 2016;58(5):514-28.<br>• Lovell AR, Ernst ME. Drug-Induced Hypertension: Focus on Mechanisms and Management. Current Hypertension Reports. 2017;19(5):39.	• The expert panel emphasized the occurence of "uncontrolled hypertension" and the need to evaluate antidepressant use, particularly when other potential triggers have been ruled out.
**B. Orthostatic hypotension/dizziness**		
10. Prescribed TCA (in doses ≥ 50 mg/day)[1] or trazodone or tranylcypromine[2]<br>- and patient has developed persistent OH/dizziness under treatment.<br><br>11. Prescribed SSRI or SNRI or mirtazapine<br>- and patient is aged ≥65 years and has developed persistent OH/dizziness under treatment.	• Bhanu C, Nimmons D, Petersen I, Orlu M, Davis D, Hussain H, et al. Drug-induced orthostatic hypotension: A systematic review and meta-analysis of randomised controlled trials. PLOS Medicine. 2021;18(11):e1003821.<br>• Calvi A, Fischetti I, Verzicco I, Belvederi Murri M, Zanetidou S, Volpi R, et al. Antidepressant Drugs Effects on Blood Pressure. Frontiers in cardiovascular medicine. 2021;8:704281.	

12. Prescribed TCA (in doses ≥ 50 mg/day)[1] or trazodone or tranylcypromine[2]<br>- and patient is aged ≥65 years and co-prescribed ≥1 further drug with known blood pressure lowering effect (e.g., α-blockers, β-blockers, nitrates, SGLT-inhibitors, levodopa, antipsychotics)[6].	• Rivasi G, Rafanelli M, Mossello E, Brignole M, Ungar A. Drug-Related Orthostatic Hypotension: Beyond Anti-Hypertensive Medications. Drugs & aging. 2020;37(10):725-38.<br>• Carvalho AF, Sharma MS, Brunoni AR, Vieta E, Fava GA. The Safety, Tolerability and Risks Associated with the Use of Newer Generation Antidepressant Drugs: A Critical Review of the Literature. Psychotherapy and Psychosomatics. 2016;85(5):270-88.<br>• Teply RM, Packard KA, White ND, Hilleman DE, DiNicolantonio JJ. Treatment of Depression in Patients with Concomitant Cardiac Disease. Progress in Cardiovascular Diseases. 2016;58(5):514-28.	• Due to the high prevalence of orthostatic hypotension (OH) in geriatric patients, which can lead to falls and fractures, it was agreed upon by the experts that the use of listed antidepressants should be reviewed even in the absence of current OH or dizziness when combined with other drugs known to lower blood pressure.
13. Prescribed SSRI or SNRI or mirtazapine<br>- and patient is aged ≥65 years and co-prescribed ≥2 further drugs with blood pressure lowering effect (e.g., α-blockers, β-blockers, nitrates, SGLT-inhibitors, levodopa, antipsychotics)[6].<br>[6]Examples of other drugs with known blood pressure lowering effect are listed in the references in this section.		
**C. Falls and fall-related injuries**		
14. Prescribed any antidepressant<br>- and patient is aged ≥65 years and co-prescribed ≥1 further fall risk-increasing drug.[7]<br><br>15. Prescribed any antidepressant<br>- and patient has a history of fall.<br><br>16. Prescribed any antidepressant<br>- and patient has cognitive impairment or dementia.<br><br>17. Prescribed any antidepressant<br>- and patients has a history of stroke and co-prescribed ≥1 further fall risk-increasing drug.[7]	• Seppala LJ, Wermelink A, de Vries M, Ploegmakers KJ, van de Glind EMM, Daams JG, et al. Fall-Risk-Increasing Drugs: A Systematic Review and Meta-Analysis: II. Psychotropics. Journal of the American Medical Directors Association. 2018;19(4):371.e11-.e17.<br>• Seppala LJ, Petrovic M, Ryg J, Bahat G, Topinkova E, Szczerbińska K, et al. STOPPFall (Screening Tool of Older Persons Prescriptions in older adults with high fall risk): a Delphi study by the EuGMS Task and Finish Group on Fall-Risk-Increasing Drugs. Age and ageing. 2021;50(4):1189-99.<br>• van Poelgeest EP, Pronk AC, Rhebergen D, van der Velde N. Depression, antidepressants and fall risk: therapeutic dilemmas-a clinical review. European geriatric medicine. 2021;12(3):585-96.<br>• van Doorn C, Gruber-Baldini AL, Zimmerman S, Hebel JR, Port CL, Baumgarten M, et al. Dementia as a risk factor for falls and fall injuries among nursing home residents. Journal of the American Geriatrics Society. 2003;51(9):1213-8.<br>• Jones JS, Kimata R, Almeida OP, Hankey GJ. Risk of Fractures in Stroke Patients Treated With a Selective Serotonin Reuptake Inhibitor: A	• Several antidepressant adverse effects such as sedation, impaired balance/reaction time, orthostatic hypotension, dizziness, cardiac conduction abnormalities, arrhythmias, drug-induced movement disorders, insomnia, and hyponatremia may contribute to or cause falls in the elderly. Differentiating the risk among different antidepressants, particularly in patients with fall risk factors such as history of falls, cognitive impairment/dementia, or stroke history is challenging.

[7]See STOPPFall for a complete list of fall-risk increasing drugs.	Systematic Review and Meta-Analysis. Stroke. 2021 Aug;52(9):2802-2808.<br>• American Geriatrics Society 2019 Updated AGS Beers Criteria® for Potentially Inappropriate Medication Use in Older Adults. Journal of the American Geriatrics Society. 2019;67(4):674-94.	
**D. Cognitive decline & Delirium**		
18. Prescribed anticholinergic antidepressant opipramol or other TCA (in doses ≥50 mg/day)[1] or paroxetine<br>- and patient has cognitive impairment or dementia.	• Mintzer J, Burns A. Anticholinergic side-effects of drugs in elderly people. J R Soc Med 2000; 93 (9):457-62.<br>• O'Mahony D, Cherubini A, Guiteras AR, Denkinger M, Beuscart JB, Onder G, Gudmundsson A, Cruz-Jentoft AJ, Knol W, Bahat G, van der Velde N, Petrovic M, Curtin D. STOPP/START criteria for potentially inappropriate prescribing in older people: version 3. Eur Geriatr Med. 2023 Aug;14(4):625-632.<br>• Kiesel EK, Hopf YM, Drey M. An anticholinergic burden score for German prescribers: score development. BMC Geriatrics. 2018;18(1):239.	• The evidence on antidepressants other than TCAs causing cognitive decline is limited. However, experts agreed to include all anticholinergic antidepressants with ACB Score 2 (opipramol and paroxetine) and ACB Score 3 (tricyclic antidepressants) in the assessment.<br>• Although a higher anticholinergic burden (ACB Score of 4 or ≥5) may be linked to an increased risk of adverse outcomes, including cognitive decline and delirium, the expert panel recommended considering a reduction in ACB score once a score of 3 is reached. This is especially relevant due to the common co-prescription of multiple low-potency anticholinergic medications.<br>• The inclusion of ACB Score thresholds in the indicator was omitted as its use in primary care setting is limited.
19. Prescribed anticholinergic antidepressant opipramol or other TCA (in doses ≥ 50 mg/day)[1] or paroxetine<br>- and patients has a history of delirium and co-prescribed ≥1 further drug known to induce delirium (e.g., benzodiazepines, opioids, antihistamines, diuretics)[8].<br><br>20. Prescribed anticholinergic antidepressant opipramol or other TCA (in doses ≥ 50 mg/day)[1] or paroxetine<br>- and patient is aged ≥65 years and co-prescribed ≥2 further drugs known to induce delirium (e.g.,	• Alagiakrishnan K, Wiens CA. An approach to drug induced delirium in the elderly. Postgraduate Medical Journal 2004;80:388-393.<br>• Clegg A, Young JB. Which medications to avoid in people at risk of delirium: a systematic review. Age Ageing. 2011 Jan;40(1):23-9.<br>• Iglseder B et al. Medikamenten-induzierte Delirien älterer Menschen [Drug-related delirium in elderly patients]. Wien Med Wochenschr. 2010; 160(11-12):281-285	• The evidence on antidepressants other than TCAs causing delirium is limited. However, experts agreed to include all anticholinergic antidepressants with ACB Score 2 (opipramol and paroxetine) and ACB Score 3 (tricyclic antidepressants) in the assessment.

benzodiazepines, opioids, antihistamines, diuretics)[8].<br><br>[8]Examples of other drugs known to induce delirium are listed in the references in this section.		
**E. Serotonin syndrome**		
21. Prescribed tranylcypromine[2]<br>- and patient is co-prescribed ≥1 further serotonergic drug (e.g., tramadol, fentanyl, triptans, metoclopramide, SSRI, SNRI, TCA)[9].<br><br>22. Prescribed SSRI or SNRI or TCA (particularly clomipramine and imipramine) (in doses ≥ 50 mg/day)[1]<br>- and patient is co-prescribed ≥2 further serotonergic drugs other than tranylcypromine (e.g. tramadol, fentanyl, triptans, metoclopramide, another serotonergic antidepressant)[9].<br><br>[9]Examples of other drugs known to induce serotonin syndrome are listed in the references in this section.	• Bartlett D. Drug-Induced Serotonin Syndrome. Crit Care Nurse. 2017 Feb;37(1):49-54. doi: 10.4037/ccn2017169. PMID: 28148614.<br>• Foong AL, Grindrod KA, Patel T, Kellar J. Demystifying serotonin syndrome (or *serotonin toxicity*). Can Fam Physician. 2018 Oct;64(10):720-727.<br>• Francescangeli J, Karamchandani K, Powell M, Bonavia A. The Serotonin Syndrome: From Molecular Mechanisms to Clinical Practice. Int J Mol Sci. 2019 May 9;20(9):2288.<br>• Scotton WJ, Hill LJ, Williams AC, Barnes NM. Serotonin Syndrome: Pathophysiology, Clinical Features, Management, and Potential Future Directions. Int J Tryptophan Res. 2019 Sep 9;12:1178646919873925..<br>• Christina Sun-Edelstein, Stewart J Tepper & Robert E Shapiro (2008) Drug induced serotonin syndrome: a review, Expert Opinion on Drug Safety, 7:5, 587-596.	• The available evidence on serotonin syndrome is limited due to its infrequent diagnosis, making it uncertain which drugs have the highest risk of causing serotonin syndrome.<br>• Based on expert consensus (3rd round ratings), SSRIs, SNRIs, and TCAs (particularly clomipramine and imipramine) were consented as antidepressants with a comparable risk of inducing serotonin syndrome.
**F. Gastrointestinal bleeding**		
23. Prescribed SSRI or SNRI<br>- and patient is aged ≥65 years and co-prescribed a single of the following without GI-protection: antiplatelet, anticoagulant, NSAID.<br><br>24. Prescribed SSRI or SNRI<br>- and patient is aged ≥65 years and co-prescribed ≥2 of the following: antiplatelet, anticoagulant, NSAID (regardless of GI-protection).	• Jiang HY, Chen HZ, Hu XJ, Yu ZH, Yang W, Deng M, et al. Use of selective serotonin reuptake inhibitors and risk of upper gastrointestinal bleeding: a systematic review and meta-analysis. Clinical gastroenterology and hepatology : the official clinical practice journal of the American Gastroenterological Association. 2015;13(1):42-50.e3.<br>• Nochaiwong S, Ruengorn C, Awiphan R, Chai-Adisaksopha C, Tantraworasin A, Phosuya C, et al. Use of serotonin reuptake inhibitor antidepressants and the risk of bleeding complications in	• The association between TCAs and gastrointestinal bleeding has been poorly investigated. Therefore, only SSRIs and SNRIs were listed as antidepressants with a considerable risk of gastrointestinal bleeding, especially when other risk factors are present (e.g., concurrent use of other medications).

25. Prescribed SSRI or SNRI<br>- and patient has at least one risk factor for GI bleeding (history of peptic ulcer disease, GI-bleeding or haemophilia) and co-prescribed ≥1 of the following: antiplatelet, anticoagulant, NSAID (regardless of GI-protection).	patients on anticoagulant or antiplatelet agents: a systematic review and meta-analysis. Annals of medicine. 2022;54(1):80-97.<br>• Anglin R, Yuan Y, Moayyedi P, Tse F, Armstrong D, Leontiadis GI. Risk of upper gastrointestinal bleeding with selective serotonin reuptake inhibitors with or without concurrent nonsteroidal anti-inflammatory use: a systematic review and meta-analysis. The American journal of gastroenterology. 2014;109(6):811-9.<br>• Alam SM, Qasswal M, Ahsan M, Walters R, Chandra S. S639 Selective Serotonin Reuptake Inhibitors Increase Risk of Upper Gastrointestinal Bleeding When Used with NSAIDs: A Systemic Review and Meta-Analysis. 2021;116:S290.<br>• Yuet WC, Derasari D, Sivoravong J, Mason D, Jann M. Selective Serotonin Reuptake Inhibitor Use and Risk of Gastrointestinal and Intracranial Bleeding. J Am Osteopath Assoc. 2019 Feb 1;119(2):102-111.<br>• Laporte S, Chapelle C, Caillet P, Beyens MN, Bellet F, Delavenne X, Mismetti P, Bertoletti L. Bleeding risk under selective serotonin reuptake inhibitor (SSRI) antidepressants: A meta-analysis of observational studies. Pharmacol Res. 2017 Apr;118:19-32.	
**G. Bleeding**		
26. Prescribed SSRI<br>- and patient has a history of bleeding event and co-prescribed ≥1 of the following: anticoagulant or antiplatelet.	• Douros A, Ades M, Renoux C: Risk of intracranial hemorrhage associated with the use of antidepressants inhibiting serotonin reuptake: a systematic review. CNS Drugs 2018; 32: 321-334.<br>• Jensen MP, Ziff OJ, Banerjee G et al.: The impact of selective serotonin reuptake inhibitors on the risk of intracranial haemorrhage: A systematic review and meta-analysis. Eur Stroke J 2019; 4: 144-152.<br>• Renoux C, Vahey S, Dell'Aniello S et al.: Association of selective serotonin reuptake inhibitors with the risk for spontaneous intracranial hemorrhage. JAMA Neurology 2017; 74: 173-180.<br>• McFarland D, Merchant D, Khandai A, Mojtahedzadeh M, Ghosn O, Hirst J, Amonoo H, Chopra D, Niazi S, Brandstetter J, Gleason A, Key G, di Ciccone BL. Selective Serotonin Reuptake Inhibitor (SSRI) Bleeding Risk: Considerations for the Consult-Liaison Psychiatrist. Curr Psychiatry Rep. 2023 Mar;25(3):113-124.	
27. Prescribed SSRI<br>- and patient has at least one risk factor for intracranial bleeding (aged ≥ 65 years, history of stroke, history of dementia) and co-prescribed ≥1 of the following: anticoagulant or antiplatelet.		• Expert panel participants agreed that concurrent treatment with oral anticoagulants and antiplatelet agents should be reserved for exceptional cases due to the significantly increased risk of bleeding. Consequently, the threshold value for indicator Nr. 27 was adjusted to include comedication with at least one of the following: anticoagulant or antiplatelet.<br>• The HAS-BLED score identifies age and history of stroke as risk factors for bleeding, while dementia increases the risk of spontaneous bleeding due to central nervous system parenchymal disturbance.

	• Waziry R, Chibnik LB, Bos D, Ikram MK, Hofman A. Risk of hemorrhagic and ischemic stroke in patients with Alzheimer disease: A synthesis of the literature. Neurology. 2020 Feb 11;94(6):265-272.<br>• Zeng Z, Chen J, Qian J, Ma F, Lv M, Zhang J. Risk Factors for Anticoagulant-Associated Intracranial Hemorrhage: A Systematic Review and Meta-analysis. Neurocrit Care. 2023 Jan 20.<br>• Rahman AA, He N, Rej S, Platt RW, Renoux C. Concomitant Use of Selective Serotonin Reuptake Inhibitors and Oral Anticoagulants and Risk of Major Bleeding: A Systematic Review and Meta-analysis. Thromb Haemost. 2023 Jan;123(1):54-63.	
**H. Constipation**		
28. Prescribed anticholinergic antidepressant opipramol or other TCA (in doses ≥ 50 mg/day)[1] or paroxetine<br>- and patient has persistent constipation.<br><br>29. Prescribed anticholinergic antidepressant opipramol or other TCA (in doses ≥ 50 mg/day)[1] or paroxetine<br>- and patient is aged ≥ 65 years and co-prescribed ≥2 further drugs known to have constipating effects (e.g., calcium antagonists, opioid, antihistamines, antipsychotics).	• O'Mahony D, Cherubini A, Guiteras AR, Denkinger M, Beuscart JB, Onder G, Gudmundsson A, Cruz-Jentoft AJ, Knol W, Bahat G, van der Velde N, Petrovic M, Curtin D. STOPP/START criteria for potentially inappropriate prescribing in older people: version 3. Eur Geriatr Med. 2023 Aug;14(4):625-632.<br>• Kiesel EK, Hopf YM, Drey M. An anticholinergic burden score for German prescribers: score development. BMC Geriatrics. 2018;18(1):239.<br>• Branch RL, Butt TF. Drug-induced constipation. Adverse Drug Reaction Bulletin. 2009(257).	• All anticholinergic antidepressants with ACB Score 2 (opipramol and paroxetine) and ACB Score 3 (tricyclic antidepressants) were included in the assessment.
**I. Hyponatremia**		
30. Prescribed any antidepressant<br>- and patient has developed hyponatremia (<130 mmol/l) under treatment without being treated with a diuretic.	• De Picker, L., Van Den Eede, F., Dumont, G., Moorkens, G., & Sabbe, B. G. (2014). Antidepressants and the risk of hyponatremia: a class-by-class review of literature. Psychosomatics, 55(6), 536-547.<br>• Greenblatt, H. Karl; Greenblatt, David J.. Antidepressant-Associated Hyponatremia in the Elderly. Journal of Clinical Psychopharmacology 36(6):p 545-549, December 2016.	• Evidence on which antidepressant is not associated with hyponatremia is missing. Nearly all antidepressants have been reported to be linked to hyponatremia. Therefore, differentiation between antidepressants when hyponatremia occurs during treatment without diuretic use was not justified.

31. Prescribed SSRI or SNRI<br>- and patient is aged ≥ 65 years and co-prescribed ≥2 further drugs known to cause hyponatremia (e.g., (thiazide) diuretics, antipsychotics, anticonvulsants, proton pump inhibitors)[10].<br><br>[10]Examples of other drugs known to cause hyponatremia are listed in the references in this section.	• Leth-Møller KB, Hansen AH, Torstensson M, Andersen SE, Ødum L, Gislasson G, et al. Antidepressants and the risk of hyponatremia: a Danish register-based population study. 2016;6(5):e011200.<br>• Mazhar F, Pozzi M, Gentili M, Scatigna M, Clementi E, Radice S, et al. Association of Hyponatraemia and Antidepressant Drugs: A Pharmacovigilance–Pharmacodynamic Assessment Through an Analysis of the US Food and Drug Administration Adverse Event Reporting System (FAERS) Database. CNS drugs. 2019;33(6):581-92.<br>• Takeda K, Kobayashi C, Nakai T, Oishi T, Okada A. Analysis of the Frequency and Onset Time of Hyponatremia/Syndrome of Inappropriate Antidiuretic Hormone Induced by Antidepressants or Antipsychotics. The Annals of pharmacotherapy. 2022;56(3):303-8.<br>• Lien, Y. H. H. (2018). Antidepressants and hyponatremia. Am J Med, 131(1), 7-8.<br>• Pinkhasov A, Xiong G, Bourgeois JA, Heinrich TW, Huang H, Coriolan S, et al. Management of SIADH-related hyponatremia due to psychotropic medications - An expert consensus from the Association of Medicine and Psychiatry. Journal of psychosomatic research. 2021;151:110654.<br>• Seifert, J., Letmaier, M., Greiner, T. et al. Psychotropic drug-induced hyponatremia: results from a drug surveillance program–an update. J Neural Transm 128, 1249–1264 (2021).<br>• Taylor, D. M., Barnes, T. R. E., & Young, A. H. (2018). The Maudsley prescribing guidelines in psychiatry (13th ed.). John Wiley & Sons.	• Hyponatremia due to medication is common in the elderly and associated with significant consequences, such as a high risk of falls, cognitive impairment, and hospitalization.<br>• Regardless of the fact that the common outcome of a critical review might be the measurement of sodium levels and not discontinuation of SSRI or SNRI, it is important to identify asymptomatic patients with hyponatremia during antidepressant treatment to prevent potential harm.
**J. Hepatic injury**		
32. Prescribed agomelatine<br>- and patient has developed elevated serum transaminase levels (>3 times the upper normal range) under treatment.<br><br>33. Prescribed agomelatine<br>- and patient has hepatic impairment (i.e. cirrhosis or active liver disease).	• Freiesleben SD, Furczyk K. A systematic review of agomelatine-induced liver injury. Journal of molecular psychiatry. 2015;3(1):4.<br>• Voican CS, Corruble E, Naveau S, Perlemuter G. Antidepressant-induced liver injury: a review for clinicians. The American journal of psychiatry. 2014;171(4):404-15.<br>• Khawagi WY, Steinke DT, Nguyen J, Pontefract S, Keers RN. Development of prescribing safety indicators related to mental health disorders and medications: Modified e-Delphi study. British journal of clinical pharmacology. 2021;87(1):189-209.	

**K. Voiding disorders**		
34. Prescribed anticholinergic antidepressant opipramol or other TCA (in doses ≥ 50 mg/day)[1] or paroxetine<br>- and patient has a history of voiding disorders (e.g., urinary retention or benign prostatic hyperplasia) or has developed urinary retention under treatment.	• Mintzer J, Burns A. Anticholinergic side-effects of drugs in elderly people. J R Soc Med 2000; 93 (9):457-62.<br>• O'Mahony D, Cherubini A, Guiteras AR, Denkinger M, Beuscart JB, Onder G, Gudmundsson A, Cruz-Jentoft AJ, Knol W, Bahat G, van der Velde N, Petrovic M, Curtin D. STOPP/START criteria for potentially inappropriate prescribing in older people: version 3. Eur Geriatr Med. 2023 Aug;14(4):625-632.<br>• Kiesel EK, Hopf YM, Drey M. An anticholinergic burden score for German prescribers: score development. BMC Geriatrics. 2018;18(1):239.	• See comments for indicator 18 & 19.
**L. Glaucoma**		
35. Prescribed anticholinergic antidepressant opipramol or other TCA (in doses ≥ 50 mg/day)[1] or paroxetine<br>- and patient has a history of angle closure glaucoma or has developed angle closure glaucoma under treatment.	• Mintzer J, Burns A. Anticholinergic side-effects of drugs in elderly people. J R Soc Med 2000; 93 (9):457-62.<br>• O'Mahony D, Cherubini A, Guiteras AR, Denkinger M, Beuscart JB, Onder G, Gudmundsson A, Cruz-Jentoft AJ, Knol W, Bahat G, van der Velde N, Petrovic M, Curtin D. STOPP/START criteria for potentially inappropriate prescribing in older people: version 3. Eur Geriatr Med. 2023 Aug;14(4):625-632.<br>• Kiesel EK, Hopf YM, Drey M. An anticholinergic burden score for German prescribers: score development. BMC Geriatrics. 2018;18(1):239.	• See comments for indicator 18 & 19.
**M. Sleep disturbances/agitation**		
36. Prescribed SSRI or SNRI or MAOI or bupropion<br>- and patient has persistent sleeping disturbances (e.g., insomnia, restless leg syndrome) or is experiencing agitation.	• Carvalho AF, Sharma MS, Brunoni AR, Vieta E, Fava GA. The Safety, Tolerability and Risks Associated with the Use of Newer Generation Antidepressant Drugs: A Critical Review of the Literature. Psychotherapy and Psychosomatics. 2016;85(5):270-88.	

	• Wichniak A, Wierzbicka A, Walęcka M, Jernajczyk W. Effects of Antidepressants on Sleep. Curr Psychiatry Rep. 2017 Aug 9;19(9):63.<br>• Kolla BP, Mansukhani MP, Bostwick JM. The influence of antidepressants on restless legs syndrome and periodic limb movements: A systematic review. Sleep medicine reviews. 2018;38:131-40.<br>• Alberti S, Chiesa A, Andrisano C, Serretti A. Insomnia and somnolence associated with second-generation antidepressants during the treatment of major depression: a meta-analysis. J Clin Psychopharmacol. 2015 Jun;35(3):296-303.
**N. Sexual dysfunction**	
37. Prescribed SSRI or SNRI<br>- and patient has developed sexual dysfunction.	• Rothmore J. Antidepressant-induced sexual dysfunction. Med J Aust. 2020 Apr;212(7):329-334.<br>• Winter J, Curtis K, Hu B, Clayton AH. Sexual dysfunction with major depressive disorder and antidepressant treatments: impact, assessment, and management. Expert Opin Drug Saf. 2022 Jul;21(7):913-930.<br>• Reichenpfader U, Gartlehner G, Morgan LC, Greenblatt A, Nussbaumer B, Hansen RA, et al. Sexual dysfunction associated with second-generation antidepressants in patients with major depressive disorder: results from a systematic review with network meta-analysis. Drug safety. 2014;37(1):19-31.
