# Supplementary material for: Deprescribing of antidepressants: development of indicators of high-risk and overprescribing using the RAND/UCLA Appropriateness Method
Source: BMC Med. 2024 May 13;22:193. doi: 10.1186/s12916-024-03397-w (PMC11089726; doi:10.1186/s12916-024-03397-w)
Supplement: Supplementary file 4 — Additional file 4. Expert ratings of round two of the RAM-Survey for overprescribing. [file 12916_2024_3397_MOESM4_ESM.pdf]

DEPRESCRIBING OF ANTIDEPRESSANTS: DEVELOPMENT OF INDICATORS OF HIGH-RISK AND OVERPRESCRIBING USING THE RAND/UCLA APPROPRIATENESS METHOD

Additional file 4: Expert ratings of round two of the RAM-Survey for overprescribing

eTable 4: Expert ratings of round two of the RAM-Survey for overprescribing

<u>Indicators of potential overprescribing</u>

	Necessity to review			
	Nr. of experts rating: Not necessary (1-3)	Nr. of experts rating: Might be necessary (4-6)	Nr. of experts rating: Clearly necessary (7-9)	Median
**Indication 1: Use of antidepressants for treating depression**				
1. First episode of a mild depression prescribed an antidepressant.	2	1	7	8
2. First episode of a moderate depression prescribed an antidepressant.	4	4	2	4
3. First episode of a mild or moderate depression and co-prescribed two antidepressants.	0	1	9	8
4. "Aged < 65 years, prescribed an antidepressant for ≥ 4 weeks, but < 8 weeks and no signs of clinically relevant symptom improvement*. *no clinically relevant symptom improvement: reduction of depressive symptoms by less than 50% of baseline at treatment initiation"	1	1	8	7
5. Aged < 65 years, prescribed an antidepressant for ≥ 8 weeks, but < 12 weeks and no signs of clinically relevant symptom improvement.	1	0	9	9
6. Aged < 65 years, prescribed an antidepressant for ≥ 12 weeks and no signs of clinically relevant symptom improvement.	0	1	9	9
7. Aged ≥ 65 years, prescribed an antidepressant for ≥ 6 weeks, but < 12 weeks and no signs of clinically relevant symptom improvement.	1	1	8	7
8. Aged ≥ 65 years, prescribed an antidepressant for ≥ 12 weeks, but < 18 weeks and no signs of clinically relevant symptom improvement.	1	0	9	9
9. Aged ≥ 65 years, prescribed an antidepressant for ≥ 18 weeks and no signs of clinically relevant symptom improvement.	0	0	10	9
10. First episode of a mild depression prescribed an antidepressant ≥ 6 months, but < 9 months (total duration of therapy).	2	6	2	5
11. First episode of a mild depression prescribed an antidepressant ≥ 9 months, but < 12 months (total duration of therapy).	0	2	8	7
12. First episode of a mild depression prescribed an antidepressant ≥ 12 months (total duration of therapy).	0	0	10	9
13. First episode of a moderate depression prescribed an antidepressant ≥ 9 months, but < 12 months after full remission.	2	5	3	5
14. First episode of a moderate depression prescribed an antidepressant ≥ 12 months, but < 15 months after full remission.	1	2	7	7
15. First episode of a moderate depression prescribed an antidepressant ≥ 15 months after full remission.	1	1	8	8
16. First episode of severe depression and prescribed an antidepressant ≥ 9 months but < 12 months after full remission.	5	5	0	4
17. First episode of severe depression and prescribed an antidepressant ≥ 12 months but < 15 months after full remission.	3	5	2	6
18. First episode of severe depression and prescribed an antidepressant ≥ 15 months but < 18 months after full remission.	2	2	6	7
19. First episode of severe depression and prescribed an antidepressant ≥ 18 months but < 24 months after full remission.	1	3	6	8
20. First episode of severe depression and prescribed an antidepressant ≥ 24 months after full remission.	1	2	7	8
21. History of 2 or more depressive episodes in the past and prescribed an antidepressant ≥ 2 years and < 3 years after full remission.	3	3	4	6
22. History of 2 or more depressive episodes in the past and prescribed an antidepressant ≥ 3 years after full remission.	2	2	6	9
23. Prescribed SSRI at a dose of > 1 DDD but ≤ 1,5 DDD following no clinically relevant symptom improvement under an SSRI dose ≤ 1 DDD.	0	3	7	9
24. Prescribed SSRI at a dose of > 1,5 DDD following no clinically relevant symptom improvement under an SSRI dose ≤ 1 DDD.	0	2	8	8
25. Prescribed two antidepressants, none of which is Mirtazapin or Mianserin.	0	3	7	7
26. Co-prescribed Mirtazapin and one of the following antidepressants (SSRI/SNRI/TZA) ≥ 9 months but < 12 months.	1	3	6	7
27. Co-prescribed Mirtazapin and one of the following antidepressants (SSRI/SNRI/TZA) ≥ 12 months and < 15 months.	1	2	7	7

28. Co-prescribed Mirtazapin and one of the following antidepressants (SSRI/SNRI/TZA) ≥ 15 months and < 18 months.	1	1	8	8
29. Co-prescribed Mirtazapin and one of the following antidepressants (SSRI/SNRI/TZA) ≥ 18 months and < 24 months.	1	0	9	8
30. Co-prescribed Mirtazapin and one of the following antidepressants (SSRI/SNRI/TZA) ≥ 24 months.	1	0	9	9
**Indications 2: Use of antidepressants for treating anxiety (e.g. panic disorder, generalized anxiety disorder or social anxiety disorder)**				
31. Anxiety and prescribed an antidepressant ≥ 6 months but < 12 months after full remission.	4	3	3	6
32. Anxiety and prescribed an antidepressant ≥ 12 months but < 18 months after full remission.	3	0	7	7
33. Anxiety and prescribed an antidepressant ≥ 18 months but < 24 months after full remission.	2	0	8	8
34. Anxiety and prescribed an antidepressant ≥ 24 months after full remission.	1	1	8	9
35. "Aged < 65 years, prescribed an antidepressant for ≥ 4 weeks, but < 8 weeks and no signs of clinically relevant symptom improvement*.*no clinically relevant symptom improvement: reduction of symptoms by less than 50% of baseline at treatment initiation"	4	4	2	5
36. Aged < 65 years, prescribed an antidepressant for ≥ 8 weeks, but < 12 weeks and no signs of clinically relevant symptom improvement.	1	2	7	8
37. Aged < 65 years, prescribed an antidepressant for ≥ 12 weeks and no signs of clinically relevant symptom improvement.	0	1	9	9
38. Aged ≥ 65 years, prescribed an antidepressant for ≥ 6 weeks, but < 12 weeks and no signs of clinically relevant symptom improvement.	4	4	2	5
39. Aged ≥ 65 years, prescribed an antidepressant for ≥ 12 weeks, but < 18 weeks and no signs of clinically relevant symptom improvement.	0	3	7	8
40. Aged ≥ 65 years, prescribed an antidepressant for ≥ 18 weeks and no signs of clinically relevant symptom improvement.	0	0	10	9
41. Anxiety and co-prescribed an antidepressant and benzodiazepine ≥ 4 weeks but < 8 weeks.	2	0	8	9
42. Anxiety and co-prescribed an antidepressant and benzodiazepine ≥ 8 weeks, but < 12 weeks.	0	2	8	9
43. Anxiety and co-prescribed an antidepressant and benzodiazepine ≥ 12 weeks.	0	0	10	9
**Indication 3: Use of antidepressants for treating insomnia**				
44. Insomnia without other indication for an antidepressant and prescribed a TCA ≥ 25 mg/day but < 50 mg/day (irrespective of the length of the treatment).	2	4	4	6
45. Insomnia without other indication for an antidepressant and prescribed a TCA ≥ 50 mg/day but < 75 mg/day (irrespective of the length of the treatment).	0	4	6	7
46. Insomnia without other indication for an antidepressant and prescribed a TCA ≥ 75 mg/day but < 100 mg/day (irrespective of the length of the treatment).	0	1	9	8
47. Insomnia without other indication for an antidepressant and prescribed a TCA ≥ 100 mg/day (irrespective of the length of the treatment).	0	0	10	9
48. Insomnia without other indication for an antidepressant and prescribed trazodone ≥ 25 mg/day but < 50 mg/day (irrespective of the length of the treatment).	3	4	3	6
49. Insomnia without other indication for an antidepressant and prescribed trazodone ≥ 50 mg/day but < 100 mg/day (irrespective of the length of the treatment).	2	2	6	7
50. Insomnia without other indication for an antidepressant and prescribed trazodone ≥ 100 mg/day but < 150 mg/day (irrespective of the length of the treatment).	0	2	8	8
51. Insomnia without other indication for an antidepressant and prescribed trazodone ≥ 150 mg/day (irrespective of the length of the treatment).	0	0	10	8
52. Insomnia without other indication for an antidepressant and prescribed mirtazapine ≥ 7,5mg/day but < 15mg (irrespective of the length of the treatment).	4	5	1	5
53. Insomnia without other indication for an antidepressant and prescribed mirtazapine ≥ 15mg/day but < 30mg (irrespective of the length of the treatment).	0	6	4	6
54. Insomnia without other indication for an antidepressant and prescribed mirtazapine ≥ 30 mg/day (irrespective of the length of the treatment).	0	2	8	8
55. Insomnia without other indication for an antidepressant and prescribed a sedating antidepressant ≥ 4 weeks but < 8 weeks.	3	6	1	5

56. Insomnia without other indication for an antidepressant and prescribed a sedating antidepressant ≥ 8 weeks but < 12 weeks.	0	5	5	7
57. Insomnia without other indication for an antidepressant and prescribed a sedating antidepressant ≥ 12 weeks.	0	1	9	9
**Indication 4: Use of antidepressants for treating pain**				
58. Neuropathic pain without other indication for an antidepressant and prescribed a TCA ≥ 25 mg/day but < 50 mg/day.	3	6	1	5
59. Neuropathic pain without other indication for an antidepressant and prescribed a TCA ≥ 50 mg/day but < 75 mg/day.	3	4	3	6
60. Neuropathic pain without other indication for an antidepressant and prescribed a TCA ≥ 75 mg/day but < 100 mg/day.	0	3	7	7
61. Neuropathic pain without other indication for an antidepressant and prescribed a TCA ≥ 100 mg/day.	0	0	10	9
62. Neuropathic pain without other indication for an antidepressant and prescribed venlafaxine ≥ 150 mg/day but < 225 mg/day.	1	2	7	8
63. Neuropathic pain without other indication for an antidepressant and prescribed venlafaxine ≥ 225 mg/day.	0	1	9	9
64. Neuropathic pain without other indication for an antidepressant and prescribed SSRI.	0	2	8	8
65. Neuropathic pain without other indication for an antidepressant and prescribed mirtazapine.	0	3	7	8
66. Non-specific low back pain without other indication for an antidepressant and prescribed any antidepressant.	0	1	9	8
67. Pain other than neuropathic pain, tension headache, migraine or fibromyalgia syndrome without other indication for an antidepressant and prescribed TCA or SNRI as analgesic.	0	1	9	8
**Miscellaneous**				
68. Chronic heart failure and first episode of a mild or moderate depression and prescribed any antidepressant.	1	1	8	8
69. Dementia and first episode of a mild or moderate depression and prescribed any antidepressant.	2	0	8	8
70. Aged ≥ 75 years and prescribed agomelatine.	1	1	8	8

Recommendations listing specific dosages are due to the fact that most tolerability studies have differentiated between antidepressant dosages. In the new version of the German depression guideline, the absolute dosage is less important. Instead, therapeutic drug monitoring is emphasized. However, this recommendation cannot yet be adopted for primary care due to lack of evidence.
